# *Mycoplasma pneumoniae* detections before and during the COVID-19 pandemic: results of a global survey, 2017 to 2021

**DOI:** 10.2807/1560-7917.ES.2022.27.19.2100746

**Published:** 2022-05-12

**Authors:** Patrick M Meyer Sauteur, Michael L Beeton, Søren A Uldum, Nathalie Bossuyt, Melissa Vermeulen, Katherine Loens, Sabine Pereyre, Cécile Bébéar, Darja Keše, Jessica Day, Baharak Afshar, Victoria J Chalker, Gilbert Greub, Ran Nir-Paz, Roger Dumke, ESGMAC–MyCOVID Study Team, ESGMAC–MyCOVID Study Team, Noémie Wagner, Corinne Andreutti, Philipp K. A. Agyeman, Christoph Aebi, Michael Buettcher, Lisa Kottanattu, Valeria Gaia, Frank Imkamp, Reinhard Zbinden, Christoph Berger, Anita Niederer-Loher, Florence Barbey, Adrian Egli, Hanna Schmid, Ulrich Heininger, Cihan Papan, Malte Kohns Vasconcelos, Birgit Henrich, Colin Mackenzie, Gerlinde Schneider, Mireille van Westreenen, Nelianne J. Verkaik, Annemarie M.C. van Rossum, Hanne-Dorthe Emborg, Ville Peltola, Marjo Renko, Terhi Tapiainen, Santtu Heinonen, Henrik Døllner, Fernanda Rodrigues, Minos Matsas, Eleni Kalogera, Evangelia Petridou, Ioannis Kopsidas, Theoklis E. Zaoutis, Ayelet Michael-Gayego, Kazunobu Ouchi, Ho Namkoong, Yu-Chia Hsieh, Matthias Maiwald, Liat Hui Loo, Rama Chaudhry, Larry K. Kociolek, Nadia Rodríguez, David Lorenz, Mary De Almeida

**Affiliations:** 1 Division of Infectious Diseases and Hospital Epidemiology, University Children’s Hospital Zurich, Zurich, Switzerland; 2 Microbiology and Infection Research Group, Department of Biomedical Sciences, Cardiff Metropolitan University, Cardiff, United Kingdom; 3 Department of Bacteria, Parasites and Fungi, Statens Serum Institute, Copenhagen, Denmark; 4 Epidemiology of Infectious Diseases, Sciensano, Brussels, Belgium; 5 Department of Microbiology, National Reference Centre for Respiratory Pathogens, University Hospital Antwerp, Antwerp, Belgium; 6 UMR CNRS 5234, Fundamental Microbiology and Pathogenicity, University of Bordeaux, Bordeaux, France; 7 Institute of Microbiology and Immunology, Faculty of Medicine, University of Ljubljana, Ljubljana, Slovenia; 8 Public Health England, London, United Kingdom; 9 Institute of Microbiology, University Hospital Center and University of Lausanne, Lausanne, Switzerland; 10 Faculty of Medicine, Hebrew University of Jerusalem, Jerusalem, Israel; 11 Department of Clinical Microbiology and Infectious Diseases, Hadassah Hebrew University Medical Center, Jerusalem, Israel; 12 TU Dresden, University Hospital Carl Gustav Carus, Institute of Medical Microbiology and Virology, Dresden, Germany; 13 European Society of Clinical Microbiology and Infectious Diseases (ESCMID) Study Group for Mycoplasma and Chlamydia Infections (ESGMAC) “Mycoplasma pneumoniae detections before and during the COVID-19 pandemic (MyCOVID)” Study Team members are listed under collaborators

**Keywords:** Face masks, nonpharmaceutical interventions (NPIs), personal protective equipment (PPE), pneumonia, *Mycoplasma pneumoniae*, severe acute respiratory syndrome coronavirus 2 (SARS-CoV-2)

## Abstract

**Background:**

*Mycoplasma pneumoniae* respiratory infections are transmitted by aerosol and droplets in close contact.

**Aim:**

We investigated global *M. pneumoniae* incidence after implementation of non-pharmaceutical interventions (NPIs) against COVID-19 in March 2020.

**Methods:**

We surveyed *M. pneumoniae* detections from laboratories and surveillance systems (national or regional) across the world from 1 April 2020 to 31 March 2021 and compared them with cases from corresponding months between 2017 and 2020. Macrolide-resistant *M. pneumoniae* (MRMp) data were collected from 1 April 2017 to 31 March 2021.

**Results:**

Thirty-seven sites from 21 countries in Europe, Asia, America and Oceania submitted valid datasets (631,104 tests). Among the 30,617 *M. pneumoniae* detections, 62.39% were based on direct test methods (predominantly PCR), 34.24% on a combination of PCR and serology (no distinction between methods) and 3.37% on serology alone (only IgM considered). In all countries, *M. pneumoniae* incidence by direct test methods declined significantly after implementation of NPIs with a mean of 1.69% (SD ± 3.30) compared with 8.61% (SD ± 10.62) in previous years (p < 0.01). Detection rates decreased with direct but not with indirect test methods (serology) (–93.51% vs + 18.08%; p < 0.01). Direct detections remained low worldwide throughout April 2020 to March 2021 despite widely differing lockdown or school closure periods. Seven sites (Europe, Asia and America) reported MRMp detections in one of 22 investigated cases in April 2020 to March 2021 and 176 of 762 (23.10%) in previous years (p = 0.04).

**Conclusions:**

This comprehensive collection of *M. pneumoniae* detections worldwide shows correlation between COVID-19 NPIs and significantly reduced detection numbers.

## Introduction

Non-pharmaceutical interventions (NPIs) were suggested to reduce the spread of severe acute respiratory syndrome coronavirus 2 (SARS-CoV-2) during the worldwide coronavirus disease (COVID-19) pandemic [1]. Many countries introduced NPIs in March 2020, which included physical distancing measures, personal protective measures (e.g. the use of masks, improved hand hygiene, respiratory etiquette), stay-at-home orders, school and day-care closures, closing borders and travel restrictions. The NPIs have been temporally associated with a global unprecedented suppression of influenza epidemics and other viral respiratory infections, such as respiratory syncytial virus (RSV) [2-8]. COVID-19 vaccinations were available as measures in addition to NPIs since December 2020 [9].

Data from some countries during the first months in 2020 indicated that the introduction of NPIs also coincided with a reduction in *Mycoplasma pneumoniae* detections [2,6,10]. *Mycoplasma pneumoniae* is a major bacterial cause of respiratory tract infections in children and adults [11]. These infections occur both endemically in many different climates across the world and epidemically every few years. Previous epidemics in Europe were reported in 2010–2012, 2014–2015 and 2015–2017 [12-15]. *Mycoplasma pneumoniae* is transmitted by aerosol particles and respiratory droplets through close contacts within families, schools, military bases, institutions (residential care and nursing homes, homes for cognitively disabled people etc.) and among closed communities [15-17].

Diagnostic tests for *M. pneumoniae* include nucleic acid amplification tests (NAAT) such as PCR, antigen tests and culture from respiratory specimens (direct test methods) or serology (indirect test method) with varying sensitivities and specificities [11,18,19]. Real-time PCR applications are the most commonly used approach for detection of *M. pneumoniae* in clinical settings [20]. However, real-time PCR is not yet standardised across laboratories [20], and there are no internationally defined guidelines on the requirements for *M. pneumoniae* testing and surveillance [14]. Some countries collect laboratory reports on *M. pneumoniae* detections through national reference laboratories (e.g. England), but only few countries have a national surveillance (e.g. Denmark) [14]. To our knowledge, no analysis on the *M. pneumoniae* incidence from several United Nations (UN) regions has been published so far.

In this study, we used survey data on laboratory *M. pneumoniae* testing and detection before and during the COVID-19 pandemic across the world to assess the impact of NPIs on the global incidence of *M. pneumoniae* in the first year after the implementation of NPIs. Of particular interest was the impact of children returning to schools on *M. pneumoniae* incidence while maintaining other NPIs during the course of the pandemic, as children are believed to be the main drivers of *M. pneumoniae* transmission [16] and have greater difficulty adhering to physical distancing and personal protective measures. In this context, was also analysed the proportion of females in particular because of their assumed closer vicinity with children.

## Methods

### Study design

#### Survey development

A structured survey was developed by a group of members from the European Society of Clinical Microbiology and Infectious Diseases (ESCMID) Study Group for Mycoplasma and Chlamydia Infections (ESGMAC), according to guidelines for survey research [21,22]. The survey consisted of six items, covering (i) details of the survey participant, (ii) information on laboratory and area, (iii) local information on stay-at-home orders and school closures during the first year of the pandemic, (iv) detailed information on the test method for *M. pneumoniae* detection (technique, product and company or reference), (v) *M. pneumoniae* test numbers (total tests, positive tests, positive tests by month, proportion of children/adolescents younger than 18 years and of females of any age) for the first 12-month period after the worldwide implementation of NPIs (1 April 2020 to 31 March 2021) and for the same period in the preceding 3 years (1 April 2017 to 31 March 2020), and (vi) macrolide-resistant *M. pneumoniae* (MRMp) testing and detection during the same periods. The survey was only administered in English and built in the SurveyMonkey online survey platform [23]. A pilot test was performed with 10 individuals (infectious diseases specialists and microbiologists) to ensure that the questions were understood and interpreted consistently and that collection of requested data was feasible within the survey time period. Details of the survey are shown in Supplementary Table S1.

#### Survey administration

Dissemination of the survey to invite participation was mixed-mode through societies (ESCMID, ESGMAC, International Organisation for Mycoplasmology (IOM) and national societies for infectious diseases and microbiology via newsletter or email distribution lists), social media (ESCMID, ESGMAC, IOM and personal accounts of authors), and through in-person contact to potential participants by one of the authors (P.M.M.S). Potential participants were defined as authors of publications about *M. pneumoniae* epidemiology (PubMed search terms: “Mycoplasma pneumoniae” [title] and “epidemiology” [all fields], 1 January 2000 to 30 March 2021; search results: 439), and more than 300 corresponding authors were approached via email. The email was accompanied by a one-page study description on behalf of the ESGMAC, the survey in PDF and Word format and the link to the online survey. Close attention was paid to ensure that all UN regions were represented during dissemination of the survey. Participation was voluntary and without compensation. There was no mechanism in place to acknowledge receipt of the survey if a laboratory did not provide information. Consent to publish the data and be listed as a participant was declared on the first page of the questionnaire. The survey was launched on 30 March 2021. Reminders were sent out after 4 and 6 weeks via social media and email. The survey was closed on 31 May 2021.

### Data collection

#### Quality control

Entries were included if they met the following quality control criteria for valid datasets: (i) verification of the participant, laboratory and institution via provided link and/or references in PubMed, (ii) validation of the information and/or references about the test method, and (iii) data check for multiple entries from the same institutions (double reporting), invalid or incomplete data, and inconsistent entries. In case of inconsistency or multiple entries from the same institutions, participants were contacted by email to request clarification and/or adapt entries to exclude double reporting. Criteria for de-duplication and exclusion criteria are listed in Supplementary Table S2.

#### Case definition

Because of local variation in the definition of *M. pneumoniae* infection, absence of clinical data and the difficulty to differentiate between *M. pneumoniae* infection and carriage [24], this study collated information on *M. pneumoniae* detections and not infections. A case was defined as *M. pneumoniae* detection in an individual with currently available test methods. Detailed information about microbiological detection methods (technique, product and company or reference) is listed in Table 1. A positive IgM, IgG or IgA serology was defined as antibody level above the cut-off of the test, as indicated by the manufacturer (Table 1). Participants were asked whether a positive serology was confirmed by a fourfold increase in IgG levels measured in convalescent samples (as serological gold standard for *M. pneumoniae* infection [11]).

**Table 1:** Demographic characteristics and laboratory information of participating sites, by United Nations (UN) region, global survey of *Mycoplasma pneumoniae* detections, April 2017–March 2021

UN region and country	City or region	National pandemic lockdown (days, period)^a^	School closure duration (days)^b^	Laboratory and/or system^c^	Test method (technique; product)	Company or reference	Macrolide resistance determination
**Europe**							
**Western Europe**							
France	Bordeaux	102 days (17 Mar–11 May 2020; 28 Oct–14 Dec 2020)	43	Hospital / clinical laboratory (tertiary centre)	NAAT (PCR, real-time; in-house)	[47]	Yes [48]
Switzerland	Geneva	41 days (16 Mar–26 Apr 2020)	31	Hospital / clinical laboratory (tertiary centre)	NAAT (multiplex PCR, real-time; BioGX Sample-Ready BD MAX System)	BD Diagnostics	No
	Lausanne			Hospital / clinical laboratory (secondary centre)	NAAT (multiplex PCR, microarray; FilmArray Respiratory Panel)	bioMérieux/BioFire Diagnostics	No
	Bern^d^			Hospital / clinical laboratory (tertiary centre)	NAAT (multiplex PCR, real-time; Anyplex II RB5 Detection)	Seegene Inc.	No
	Lucerne^d^			Hospital / clinical laboratory (tertiary centre)	NAAT (multiplex PCR, microarray; FilmArray Respiratory Panel)	bioMérieux/BioFire Diagnostics	No
	Bellinzona			**Surveillance system (regional; 0.4 million population)** ^e^	NAAT (multiplex PCR, microarray; FilmArray Respiratory Panel)^f^	bioMérieux/BioFire Diagnostics	No
	Zurich (A)			Hospital / clinical laboratory (tertiary centre)	NAAT (PCR, real-time; in-house)	[49]	Yes [50]
	Zurich (B)^d^			Hospital / clinical laboratory (tertiary centre)	NAAT (PCR, real-time; in-house)^g^	[49]	Yes [50]
	St. Gallen^d^			Hospital / clinical laboratory (tertiary centre)	NAAT (multiplex PCR, real-time; Allplex Respiratory Panel)	Seegene Inc.	No
	Aarau			Hospital / clinical laboratory (tertiary centre)	NAAT (multiplex PCR, microarray; FilmArray Respiratory Panel)	bioMérieux/BioFire Diagnostics	No
					ELISA^h^ (ImmunoWELL Mycoplasma IgM/IgG)	Thermo Fisher Scientific Remel Inc.	
	Basel (A)			Hospital / clinical laboratory (tertiary centre)	NAAT (multiplex PCR, microarray; FilmArray Respiratory Panel)	bioMérieux/BioFire Diagnostics	No
	Basel (B)^d^			Hospital / clinical laboratory (tertiary centre)	NAAT (multiplex PCR, microarray; FilmArray Respiratory Panel)^i^	bioMérieux/BioFire Diagnostics	No
Germany	Homburg	161 days (17 Mar–5 May 2020; 19 Dec 2020–end of survey period)	92	Hospital / clinical laboratory (tertiary centre)	NAAT (multiplex PCR, real-time; AID CAP Bac PCR Kit)	Autoimmun Diagnostika GmbH (AID)	No
					CLIA^h^ (*Mycoplasma pneumoniae* Virclia IgM/IgG Monotest)	Vircell, S.L.	
	Düsseldorf			Hospital / clinical laboratory (tertiary centre)	NAAT (PCR, real-time; in-house)	[51]	No
					ELISA^h^ (EIA Mycoplasma IgM/IgG/IgA)	DIAsource ImmunoAssays SA	
	Saxony^j^			**Surveillance system (regional; 4.1 million population)** ^k^	Combination of direct and indirect test methods (different techniques)^k^	[12]	No
Belgium	Antwerp, Leuven (national reference laboratory)	52 days (18 Mar–9 May 2020)	76	Hospital / clinical laboratory (tertiary centre) and **national reference laboratory** ^l^	NAAT (PCR, real-time; in-house)	[52]	Yes [48]
	National surveillance^j^			**Surveillance system (national; 60% of all Belgian microbiology laboratories)** ^m^	Direct test methods (different techniques)^m^	[53]	No
The Netherlands	Rotterdam	99 days (16 Mar–6 Apr 2020; 15 Dec 2020–2 Mar 2021)	74	Hospital / clinical laboratory (tertiary centre)	NAAT (PCR, real-time; in-house)	[54]	No
**Northern Europe**							
England	National reference laboratory^n^	72 days (14 Mar– 9 May 2020; 5 Nov–1 Dec 2020)	102	**National reference laboratory**	NAAT (multiplex PCR, real-time; in-house)	[20]	Yes [55]
Denmark	National surveillance	99 days (12 Mar–13 Apr 2020; 25 Dec–1 Mar 2020)	76	**Surveillance system (national; 5.8 million population)**	NAAT (PCR, different techniques)^o^	[56]	No
Finland	Turku	98 days (16 Mar–22 Jun 2020)	42	Hospital / clinical laboratory (tertiary centre)	Combination of direct and indirect test methods (different techniques)^p^	[57]	No
	National surveillance^j^			**Surveillance system (national; 5.5 million population)**	Combination of direct and indirect test methods (different techniques)^q^	[6]	No
Norway	Trondheim	81 days (12 Mar–1 Jun 2020)	32	Hospital / clinical laboratory (tertiary centre)	NAAT (multiplex PCR, real-time; in-house)	NA	No
**Southern Europe**							
Portugal	Coimbra^d^	103 days (19 Mar–2 May 2020; 15 Jan–15 Mar 2021)	67	Hospital / clinical laboratory (tertiary centre)	NAAT (multiplex PCR, microarray; FilmArray Respiratory Panel)	bioMérieux/BioFire Diagnostics	No
Greece	Athens (A)^d^	179 days (23 Mar–4 May 2020; 7 Nov 2020–22 Mar 2021)	114	Hospital / clinical laboratory (tertiary centre)	ELISA (DRG *Mycoplasma pneumoniae* ELISA IgM/IgG)	DRG International, Inc.	No
	Athens (B)^d^			Hospital / clinical laboratory (tertiary centre)	ELISA (NovaLisa *Mycoplasma pneumoniae* IgM/IgG)	Novatec Immundiagnostica GmbH	No
Slovenia	Ljubljana	46 days (19 Mar–4 May 2020)	46	Hospital / clinical laboratory (tertiary centre)	NAAT (multiplex PCR, real-time; Chla/Myco pneumo R-GENE)	bioMérieux/ARGENE	No
**Asia**							
**Western Asia**							
Israel	Jerusalem	52 days (12 Mar–3 May 2020)	139	Hospital / clinical laboratory (tertiary centre)	NAAT (PCR, real-time; in-house)	[20]	No
**Eastern Asia**							
Japan	Kurashiki City (Okayama)^d^	0 days (no national lockdown)	51	Hospital / clinical laboratory (tertiary centre)	NAAT (PCR, real-time; in-house)	[58]	Yes [58]
	Tokyo			Hospital / clinical laboratory (secondary centre)	Rapid antigen test (SAI; FUJI DRI-CHEM IMMUNO AG)	Fujifilm, Kanagawa, Japan	No
Taiwan	Taoyuan^d^	0 days (no national lockdown)	0 (no official school closures)	Hospital / clinical laboratory (tertiary centre)	NAAT (PCR, real-time; in-house)	[59]	Yes [59]
**South-eastern Asia**							
Singapore	Singapore^d^	55 days (7 Apr–1 Jun 2020)	57	Hospital / clinical laboratory (tertiary centre)	NAAT (multiplex PCR, microarray; FilmArray Respiratory Panel)	bioMérieux/BioFire Diagnostics	No
**South Asia**							
India	New Delhi	74 days (25 Mar–7 Jun 2020)	235	Hospital / clinical laboratory (tertiary centre)	ELISA (NovaLisa *Mycoplasma pneumoniae* IgM)	Novatec Immundiagnostica GmbH	NO
**America**							
**Northern America**							
United States	Chicago^d^	70 days (21 Mar–30 May 2020)	192	Hospital / clinical laboratory (tertiary centre)	NAAT (multiplex PCR, microarray; FilmArray Respiratory Panel)	bioMérieux/BioFire Diagnostics	No
**Caribbean**							
Cuba	National surveillance	240 days (20 Mar–18 Jun 2020; 1 Nov 2020–end of survey period)	121	**Surveillance system (national; 11.3 million population)**	NAAT (PCR, real-time; in-house)	[60]	Yes [60]
**Oceania**							
Australia	Darlinghurst (Sydney)	53 days (23 Mar–15 May 2020)	125	Hospital / clinical laboratory (tertiary centre)	NAAT (PCR, real-time; EasyScreen Respiratory Pathogen Detection Kit)	Genetic Signatures	No
New Zealand	Auckland	78 days (national: 23 Mar–13 May 2020; Auckland: 12–18 Aug 2020; 15–17 Feb 2021; 28 Feb–7 Mar 2021)	40	Hospital / clinical laboratory (tertiary centre)	NAAT (multiplex PCR, microarray; FilmArray Respiratory Panel)^r^	bioMérieux/BioFire Diagnostics	No

CLIA: chemiluminescent immunoassay; ELISA: enzyme-linked immunosorbent assay; Ig, immunoglobulin; NA: not available; NAAT: nucleic acid amplification test; SAI: silver amplification immunochromatography; UN: United Nations.

^a^ Stay-at-home orders for the general population (referred to as lockdown) according to an ECDC document [25] for Europe and to Wikipedia [26] for other UN regions, with adjustments made by the local participating author and considered until the end of the study period (31 March 2021).

^b^ Full and partial school closure duration in days according to [27] until 2 March 2021 (last update before end of study period).

^c^ More detailed information including reporting characteristics, de-duplication and exclusion criteria are provided in Supplementary Table S2.

^d^ ≥ 90% of data are from children and adolescents < 18 years of age.

^e^ Data from several hospitals in the region of Ticino.

^f^ Additional use of a specific in-house PCR [52].

^g^ From 12 October 2020 to the end of the survey period additional testing with the FilmArray Respiratory Panel (bioMérieux/BioFire Diagnostics).

^h^ In addition to PCR also serological data separately reported.

^i^ Multiplex PCR testing before 2020 using the Respifinder (Pathofinder), and single PCR testing over the total survey period with a specific in-house PCR, as described previously [61].

^j^ Exclusively positive test numbers (and no total test numbers) available and/or reported.

^k^ Data from the federal state of Saxony detected by the Landesuntersuchungsanstalt Sachsen based on combined direct and indirect test methods, but predominantly on serology (no information on isotypes) [12].

^l^ National reference laboratory data from the two related hospitals (Antwerp, Leuven; 86–98%) and across the country (2–14%).

^m^ Data collected through the Belgian Sentinel Network of Laboratories (SNL), a network of ca 95 microbiology laboratories (i.e. 60% of all Belgian microbiology laboratories) [53], based on direct test methods such as NAAT, antigen test, culture, microscopy, 'unknown' or 'other' (cases based on serology were excluded).

^n^ Period of enhanced surveillance from 1 October 2019 to 30 March 2020.

^o^ Different PCR assays, of which some are published [56] or commercial kits, but most are unpublished but validated in-house assays.

^p^ Predominantly by serology (ca 75%; no information on isotypes), partly by multiplex PCR (Allplex Respiratory Panel, Seegene Inc.; ca 25%).

^q^ Predominantly by PCR.

^r^ Additional use of an in-house multiplex PCR (www.labplus.co.nz).

Entries in bold signify national and/or regional surveillances.

#### Stay-at-home order and school closure periods

Periods of stay-at-home orders for the general population (referred to as lockdowns) in Europe were obtained from the Response Measures Database (RMD) of the European Centre for Disease Prevention and Control (ECDC) [25] and those in other UN regions from a collection of pandemic lockdown dates in Wikipedia [26], with adjustments made by the participants. The total duration in days until the end of the study period was calculated for each site. School closure duration in days (full and partial closure in total) was determined according to the United Nations Children’s Fund (UNICEF) global school closures database until 2 March 2021 (last update before the end of the study period) [27].

### Statistical analysis

Incidence was defined as the number of new cases over a specified period of time within a community [28]. Given the missing population denominators we were not able to report incidence rates. We compared *M. pneumoniae* detections between April 2020 and March 2021 with total numbers observed from April 2017 to March 2020. Fisher's exact test was used to compare proportions with corrections for multiple testing. Spearman rank correlation coefficient (*R*, rho) was used for analyses of correlation. All reported p values are two-tailed with statistical significance defined as p < 0.05. Data were analysed using R software (version 4.0.5) [29].

## Results

### Survey entries and detection methods

**Figure 1:**
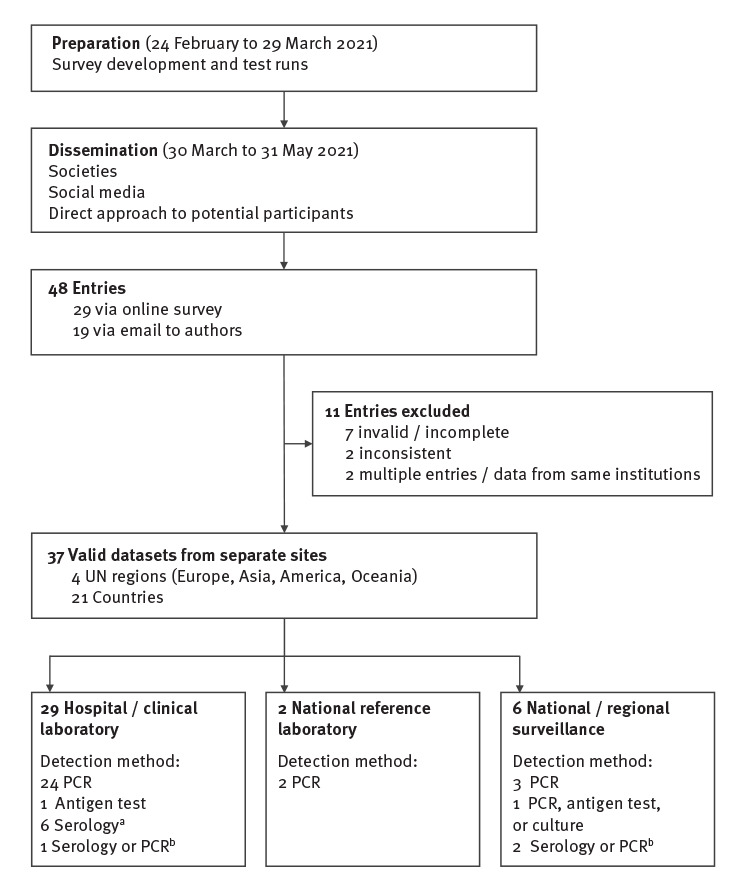
Study profile, global survey of *Mycoplasma pneumoniae* detections, April 2017–March 2021

We received entries from 48 sites, of which 29 were entered via the online survey and 19 via email to authors. Of the 12 experts collating laboratory detections of *M. pneumoniae* in Europe and Israel for the ESGMAC in a previous study (January 2011–April 2016) [14], eight provided information for this survey. An overall response rate could not be calculated because the survey was widely disseminated through societies, social media and further dissemination among participants themselves. We excluded 11 entries because of invalid or incomplete data (n = 7), inconsistent data (n = 2; positive test numbers by month did not match with total numbers per year) or double reporting (n = 2; congruent data from same institutions). Thus, 37 valid datasets from separate sites in 21 countries from four UN regions were eligible for inclusion (Europe: n = 12; Asia: n = 5; America: n = 2; Oceania: n = 2), 29 from hospital laboratories, two from national reference laboratories and six from national and/or regional surveillance systems (Figure 1).

UN: United Nations.

^a^ Three sites provided serological data in addition to PCR.

^b^ No distinction possible between detection methods, but predominantly serological data included.

Demographic characteristics and laboratory information of participating sites are shown in Table 1. The detection method varied between sites: 29 (78.38%) sites reported exclusively PCR (n = 17 multiplex); three sites used exclusively serology (enzyme-linked immunosorbent assay (ELISA)), three sites reported combined PCR and serology (no distinction possible between detection methods, but predominantly serology), one site used a combination of direct test methods (i.e. PCR, antigen test or culture) and one site used exclusively rapid antigen testing. Three sites reported only the number of positive tests over the entire study period (Saxony (Germany) and national surveillance systems of Belgium and Finland), and another three sites provided serological data in addition to PCR.

### Detections before and after the introduction of non-pharmaceutical interventions

A total of 631,104 tests were performed during the study period from April 2017–March 2021 (three sites did not have data about total test numbers available). Overall, 30,617 *M. pneumoniae* detections were confirmed from participating sites. Among those with available information on age/sex, 54.92% (n = 11,029/20,081) were reported in children/adolescents younger than 18 years of age and 52.90% (n = 12,794/24,184) in females. The greatest number of positive tests were obtained with direct test methods (n = 19,102; 62.39%; predominantly PCR) followed by a combination of PCR and serology (n = 10,483; 34.24%; no information on isotypes) or serology alone (n = 1,032; 3.37%; only IgM was considered if all isotypes were reported). Information about convalescent samples for serological testing was not available. No routine testing for a fourfold increase in IgG levels was reported. De-duplication data were determined at site level (Supplementary Table S2 lists the reporting characteristics per site).

**Figure 2:**
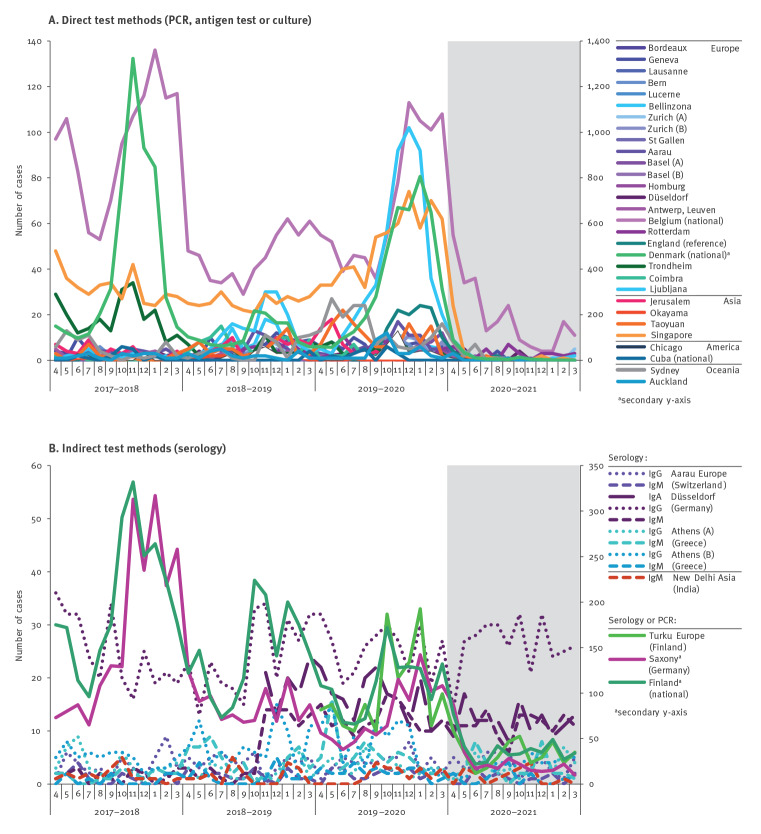
Global detection of *Mycoplasma pneumoniae*, April 2017–March 2021 (n = 30,617)

**Figure 3:**
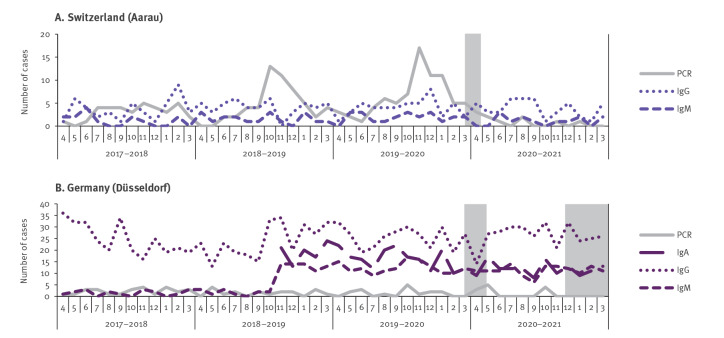
Detection of *Mycoplasma pneumoniae* at sites that provided single-sample serological data in addition to PCR, April 2017–March 2021 (n = 14,702^a^)

^a^ For serology only total test numbers of IgM considered.

There was a significant reduction of *M. pneumoniae* detections after the introduction of NPIs (Figure 2). Among total detections, 1,714 (5.60%) derived from April 2020 to March 2021 compared with 28,903 (94.40%) from April 2017 to March 2020 (Table 2). *Mycoplasma pneumoniae* testing and detection in children/adolescents and females per year is shown in Table 3. The annual proportion of children/adolescents and females with detections before and during the COVID-19 pandemic was 55.16% vs 49.77% (p < 0.01) and 53.01% vs 50.86% (p = 0.15), respectively. Detailed graphs for each site and country are shown in Supplementary Figures S1–S6. The difference in detections before and during the COVID-19 pandemic was more obvious for direct test methods (Figure 2A) than indirect test methods (Figure 2B). This is supported by a direct comparison of detections with PCR and single-sample serology (IgM, IgG and IgA) from the three sites that reported data separately for each method, which did not show any correlation between those two test methods (Figure 3).

Ig: immunoglobulin.

Data from combined serology and PCR tests are shown under indirect test methods (no distinction possible between detection methods, but predominantly serology; Table 1). For serology, only total test numbers of IgM considered. The grey backgrounds indicate the presence of non-pharmaceutical interventions during the COVID-19 pandemic. Detailed graphs separately for each site and country with corresponding local lockdown periods are shown in Supplementary Figures S1–S6.

**Table 2:** *Mycoplasma pneumoniae* testing and detection rates per year, April 2017–March 2021 (n = 631,104)

UN region and country	City or region	Test method	April 2017–March 2018			April 2018–March 2019			April 2019–March 2020			April 2020–March 2021 (COVID-19 pandemic)			Difference in detection rate (%) pre-pandemic vs COVID-19 pandemic^a^	P^b^
			Total tests (*N*)	Positive tests (*n*)	Detection rate (%)	Total tests (*N*)	Positive tests (*n*)	Detection rate (%)	Total tests (*N*)	Positive tests (*n*)	Detection rate (%)	Total tests (*N*)	Positive test (*n*)	Detection rate (%)		
**Europe**																
**Western Europe**																
France	Bordeaux	PCR	619	16	2.58	625	22	3.52	530	41	7.74	466	4	0.86	**–80.72**	**<0.01**
Switzerland	Geneva	PCR	1,347	30	2.23	1,622	76	4.69	2,119	76	3.59	1,193	7	0.59	**–83.60**	**<0.01**
	Lausanne	PCR	388	6	1.55	406	4	0.99	592	20	3.38	246	0	0.00	**–100.00**	**0.02**
	Bern^c^	PCR	134	17	12.69	175	43	24.57	191	29	15.18	41	0	0.00	**–100.00**	**<0.01**
	Lucerne^c^	PCR	NA	7	NA	229	10	4.37	215	21	9.77	129	1	0.78	**–88.90**	**<0.01**
	Bellinzona	PCR	701	10	1.43	1,104	76	6.88	1,540	43	2.79	804	0	0.00	**–100.00**	**<0.01**
	Zurich (A)	PCR	1,067	17	1.59	1,361	41	3.01	1,620	50	3.09	1,823	11	0.60	**–77.38**	**<0.01**
	Zurich (B)^c^	PCR	104	21	20.19	123	22	17.89	201	54	26.87	1,659	6	0.36	**–98.40**	**<0.01**
	St. Gallen^c^	PCR	20	7	35.00	18	5	27.78	19	6	31.58	8	1	12.50	**–60.42**	0.42
	Aarau	PCR	1,431	36	2.52	1,586	55	3.47	1,955	77	3.94	1,601	10	0.62	**–81.51**	**<0.01**
		*IgM ELISA*	*220*	*14*	*6.36*	*229*	*19*	*8.30*	*191*	*23*	*12.04*	*183*	*13*	*7.10*	**–*18.81***	*0.55*
		*IgG ELISA*	*220*	*43*	*19.55*	*229*	*50*	*21.83*	*191*	*48*	*25.13*	*183*	*46*	*25.14*	*+ 14.10*	*0.37*
	Basel (A)	PCR	1,535	9	0.59	2,212	12	0.54	5,028	53	1.05	3,061	2	0.07	**–92.25**	**<0.01**
	Basel (B)^c^	PCR	870	10	1.15	845	6	0.71	1,050	19	1.81	634	6	0.95	**–25.24**	0.69
Germany	Homburg	PCR	2,321	10	0.43	2,395	19	0.79	2,773	17	0.61	2,570	1	0.04	**–93.67**	**<0.01**
		*IgM ELISA*	*486*	*67*	*13.79*	*492*	*70*	*14.23*	*544*	*71*	*13.05*	*588*	*70*	*11.90*	**–*12.89***	*0.31*
		*IgG ELISA*	*486*	*277*	*57.00*	*492*	*291*	*59.15*	*544*	*341*	*62.68*	*588*	*331*	*56.29*	**–*5.75***	*0.15*
	Düsseldorf	PCR	1,515	27	1.78	1,530	18	1.18	1,283	16	1.25	1,011	12	1.19	**–15.79**	0.65
		*IgM ELISA*	*398*	*18*	*4.52*	*446*	*78*	*17.49*	*585*	*148*	*25.30*	*538*	*134*	*24.91*	*+ 45.87*	***<0.01***
		*IgG ELISA*	*530*	*298*	*56.23*	*491*	*288*	*58.66*	*561*	*307*	*54.72*	*522*	*315*	*60.34*	*+ 6.90*	*0.13*
		*IgA ELISA^d^*	NA	NA	NA	*241*	*95*	*39.42*	*560*	*195*	*34.82*	*521*	*142*	*27.26*	**–*24.72***	***<0.01***
	Saxony	*PCR or serology^e^*	NA	*2,013*	NA	NA	*1,044*	NA	NA	*927*	NA	NA	*303*	NA	NA	NA
Belgium	Antwerp, Leuven (national reference laboratory)	PCR	2,698	30	1.11	1,150	15	1.30	1220	32	2.62	864	3	0.35	**–77.15**	**<0.01**
	National surveillance	Direct test methods (different techniques)	NA	1,151	NA	NA	548	NA	NA	833	NA	NA	230	NA	NA	NA
The Netherlands	Rotterdam	PCR	NA	NA	NA	240	36	15.00	407	56	13.76	444	36	8.11	**–42.98**	**<0.01**
**Northern Europe**																
England	National reference laboratory^f^	PCR	138	19	13.77	110	11	10.00	263	118	44.87	155	10	6.45	**–77.72**	**<0.01**
Denmark	National surveillance	PCR	100,257	5,303	5.29	80,965	1,371	1.69	100,879	4,383	4.34	58,716	177	0.30	**–92.31**	**< 0.01**
Finland	Turku	*PCR or serology^e^*	NA	NA	NA	NA	NA	NA	*5,413*	*211*	*3.90*	*3,462*	*70*	*2.02*	**–*48.13***	***<0.01***
	National surveillance	*PCR or serology^e^*	NA	*2,420*	*NA*	*NA*	*1,728*	NA	NA	*1,312*	NA	NA	*455*	NA	NA	NA
Norway	Trondheim	PCR	3,306	230	6.96	2,330	56	2.40	2,014	48	2.38	1,263	0	0.00	**–100.00**	**<0.01**
**Southern Europe**																
Portugal	Coimbra^c^	PCR	803	5	0.62	924	90	9.74	1,084	19	1.75	161	0	0.00	**–100.00**	**<0.01**
Greece	Athens (A)^c^	*IgM ELISA*	*212*	*19*	*8.96*	*236*	*51*	*21.61*	*250*	*65*	*26.00*	*167*	*35*	*20.96*	*+ 8.36*	*0.66*
		*IgG ELISA*	*212*	*44*	*20.75*	*236*	*29*	*12.29*	*250*	*37*	*14.80*	*167*	*41*	*24.55*	*+ 55.79*	***<0.01***
	Athens (B)^c^	*IgM ELISA*	*185*	*9*	*4.86*	*181*	*15*	*8.29*	*231*	*27*	*11.69*	*172*	*14*	*8.14*	**–*4.72***	*1.00*
		*IgG ELISA*	*185*	*59*	*31.89*	*181*	*88*	*48.62*	*231*	*92*	*39.83*	*172*	*44*	*25.58*	**–*36.10***	***<0.01***
Slovenia	Ljubljana	PCR	1,604	22	1.37	1,887	153	8.11	2,639	495	18.76	1,241	20	1.61	**–85.26**	**<0.01**
**Asia**																
**Western Asia**																
Israel	Jerusalem	PCR	1,364	45	3.30	1,299	62	4.77	1,637	53	3.24	666	0	0.00	**–100.00**	**<0.01**
**Eastern Asia**																
Japan	Kurashiki City (Okayama)^c^	PCR	30	4	13.33	64	14	21.88	34	3	8.82	5	0	0.00	**–100.00**	1.00
	Tokyo^f^	Rapid antigen test	346	56	16.18	140	36	25.71	600	36	6.00	120	4	3.33	**–71.72**	**<0.01**
Taiwan	Taoyuan^c^	PCR	116	20	17.24	159	63	39.62	204	131	64.22	44	5	11.36	**–74.56**	**<0.01**
**South-eastern Asia**																
Singapore	Singapore^c^	PCR	4,212	387	9.19	8,765	307	3.50	15,860	613	3.87	8,835	33	0.37	**–91.76**	**<0.01**
**South Asia**																
India	New Delhi	*IgM ELISA*	*245*	*19*	*7.76*	*320*	*18*	*5.63*	*205*	*19*	*9.27*	*153*	*16*	*10.46*	*+ 43.79*	*0.19*
**America**																
**Northern America**																
United States	Chicago^c^	PCR	4,221	10	0.24	4,199	25	0.60	4,990	42	0.84	1,695	2	0.12	**–79.45**	**0.01**
**Caribbean**																
Cuba	National surveillance	PCR	902	18	2.00	62	4	6.45	844	20	2.37	4	0	0.00	**–100.00**	1.00
**Oceania**																
Australia	Darlinghurst (Sydney)	PCR	15,751	60	0.38	12,187	55	0.45	21,086	168	0.80	70,807	19	0.03	**–95.35**	**<0.01**
New Zealand	Auckland	PCR	543	21	3.87	993	26	2.62	858	41	4.78	2,723	4	0.15	**–96.00**	**<0.01**
**Total (global, participating countries)^g^**		**Direct test methods (PCR or rapid antigen test considered only)**	**148,343**	**6,453**	**4.35**	**129,705**	**2,733**	**2.11**	**173,735**	**6,780**	**3.90**	**162,989**	**374**	**0.23**	**–93.51**	**<0.01**
		***Indirect test methods (IgM considered only)***	***1,746***	***146***	***8.36***	***1,904***	***251***	***13.18***	***2,006***	***353***	***17.60***	***1,801***	***282***	***15.66***	*+ 18.08*	***0.01***

COVID-19: coronavirus disease; ELISA: enzyme-linked immunosorbent assay; Ig: immunoglobulin; NA: not available; UN: United Nations.

^a^ Difference in detection rate between April 2017 and March 2020 (mean positive/total tests across the 3 years) and between April 2020 and March 2021 (absolute number positive/total tests). Percentages showing a reduction in detection rate are indicated in bold.

^b^ Proportions of positive/total tests from April 2020 to March 2021 were compared with total numbers from April 2017 to March 2020 by Fisher's exact test. p values < 0.05 are indicated in bold.

^c^ ≥ 90% of data are from children and adolescents < 18 years of age.

^d^ IgA ELISA introduced in November 2018.

^e^ Data from combined serology and PCR tests (no distinction possible between detection methods; Table 1).

^f^ Period of enhanced surveillance from 1 October 2019 to 30 March 2020.

^g ^These numbers include only data from PCR or rapid antigen test (for direct test methods) and IgM serology (for indirect test methods).

Entries in italics signify serological data (± PCR).

**Table 3:** *Mycloplasma pneumoniae* testing and detection in children/adolescents and females per year, April 2017–March 2021 (n = 154,241 children/adolescents and 285,238 females)

UN region and country	City or region	Test method	April 2017–March 2018						April 2018–March 2019						April 2019–March 2020						April 2020–March 2021 (COVID-19 pandemic)					
			Children/ adolescents			Females			Children/ adolescents			Females			Children/ adolescents			Females			Children/ adolescents			Females		
			N	n	%	N	n	%	N	n	%	N	n	%	N	n	%	N	n	%	N	n	%	N	n	%
**Europe**																										
**Western Europe**																										
France	Bordeaux	PCR	335	9	2.69	236	11	4.66	282	15	5.32	280	11	3.93	272	28	10.29	248	17	6.85	220	2	0.91	193	0	0.00
Switzerland	Geneva	PCR	201	8	3.98	579	17	2.94	301	43	14.29	704	39	5.54	354	45	12.71	392	34	8.67	161	2	1.24	449	3	0.67
	Lausanne	PCR	42	1	2.38	226	5	2.21	18	1	5.56	200	1	0.50	36	4	11.11	325	9	2.77	2	0	0.00	123	0	0.00
	Bern^a^	PCR	134	17	12.69	65	8	12.31	175	43	24.57	74	18	24.32	191	29	15.18	78	14	17.95	41	0	0.00	16	0	0.00
	Lucerne^a^	PCR	NA	7	NA	NA	3	NA	229	10	4.37	NA	3	NA	215	21	9.77	NA	5	NA	129	1	0.78	NA	1	NA
	Bellinzona	PCR	155	6	3.87	315	2	0.63	471	66	14.01	500	41	8.20	354	22	6.21	661	19	2.87	118	0	0.00	328	0	0.00
	Zurich (A)	PCR	29	2	6.90	NA			43	6	13.95	NA			44	8	18.18	NA			35	1	2.86	NA		
	Zurich (B)^a^	PCR	104	21	20.19	NA			123	22	17.89	NA			201	54	26.87	NA			1,659	6	0.36	NA		
	St. Gallen^a^	PCR	20	7	35.00	14	4	28.57	18	5	27.78	12	5	41.67	19	6	31.58	7	3	42.86	8	1	12.50	4	1	25.00
	Aarau	PCR	441	13	2.95	603	14	2.32	392	22	5.61	723	24	3.32	484	26	5.37	891	38	4.26	287	4	1.39	658	6	0.91
		*IgM ELISA*	*25*	*4*	*16.00*	*91*	*10*	*10.99*	*20*	*8*	*40.00*	*99*	*7*	*7.07*	*33*	*8*	*24.24*	*77*	*10*	*12.99*	*16*	*3*	*18.75*	*69*	*9*	*13.04*
		*IgG ELISA*	*25*	*3*	*12.00*	*91*	*15*	*16.48*	*20*	*6*	*30.00*	*99*	*19*	*19.19*	*33*	*9*	*27.27*	*77*	*15*	*19.48*	*16*	*1* ^b^	*6.25*	*69*	*18*	*26.09*
	Basel (A)	PCR	4	0	0.00	644	6	0.93	5	0	0.00	937	7	0.75	9	0	0.00	2,201	25	1.14	1	0	0.00	1,251	2	0.16
	Basel (B)^a^	PCR	863	10	1.16	404	5	1.24	845	6	0.71	NA	1	NA	1,050	19	1.81	NA	NA	NA	634	6	0.95	NA	NA	NA
Germany	Homburg	PCR	53	2	3.77	NA	4	NA	75	3	4.00	NA	8	NA	111	4	3.60	NA	7	NA	88	0	0.00	NA	1	NA
		*IgM ELISA*	*NA*			*NA*			*NA*			*NA*			*NA*			*NA*			*NA*			*NA*		
		*IgG ELISA*	*NA*			*NA*			*NA*			*NA*			*NA*			*NA*			*NA*			*NA*		
	Düsseldorf	PCR	1,003	21	2.09	618	10	1.62	1,026	16	1.56	649	5	0.77	882	15	1.70	523	6	1.15	621	10	1.61	471	4	0.85
		*IgM ELISA*	*264*	*12*	*4.55*	*179*	*9*	*5.03*	*246*	*36*	*14.63*	*173*	*24*	*13.87*	*246*	*52*	*21.14*	*182*	*37*	*20.33*	*253*	*47*	*18.58*	*161*	*29*	*18.01*
		*IgG ELISA*	*307*	*168*	*54.72*	*237*	*142*	*59.92*	*255*	*141*	*55.29*	*187*	*118*	*63.10*	*226*	*98*	*43.36*	*174*	*96*	*55.17*	*238*	*132*	*55.46*	*157*	*103^b^*	*65.61*
		*IgA ELISA*	*NA*			*NA*			*120*	*36*	*30.00*	*80*	*26*	*32.50*	*226*	*37*	*16.37*	*174*	*46*	*26.44*	*237*	*17^b^*	*7.17*	*156*	*24*	*15.38*
	Saxony	*PCR or serology*	NA			NA			NA			NA			NA			NA			NA			NA		
Belgium	Antwerp, Leuven (national reference laboratory)	PCR	748	16	2.14	1,132	17	1.50	208	4	1.92	486	9	1.85	240	15	6.25	510	17	3.33	100	2	2.00	356	0	0.00
	National surveillance	Direct test methods (different techniques)	NA	740	NA	NA	639	NA	NA	362	NA	NA	285	NA	NA	493	NA	NA	433	NA	NA	86^b^	NA	NA	140^b^	NA
The Netherlands	Rotterdam	PCR	NA			NA			47	11	23.40	119	22	18.49	89	26	29.21	163	23	14.11	54	12	22.22	176	19	10.80
**Northern Europe**																										
England	National reference laboratory	PCR	39	8	20.51	63	7	11.11	34	2	5.88	45	9	20.00	84	51	60.71	102	50	49.02	58	7	12.07	49	5	10.20
Denmark	National surveillance	PCR	15,879	2,374	14.95	55,874	2,843	5.09	9,121	515	5.65	44,132	768	1.74	14,307	1,854	12.96	55,356	2,374	4.29	2,650	68	2.57	27,693	83	0.30
Finland	Turku	*PCR or serology*	*NA*			*NA*			*NA*			*NA*			*1,488*	*138*	*9.27*	*NA*			*804*	*51*	*6.34*	*NA*		
	National surveillance	*PCR or serology*	*NA*			*NA*	*1,344*	*NA*	*NA*			*NA*	*997*	*NA*	*NA*			*NA*	*699*	*NA*	*NA*			*NA*	*265*	*NA*
Norway	Trondheim	PCR	3,306	230	6.96	1,556	113	7.26	2,330	56	2.40	1,041	26	2.50	2,014	48	2.38	920	22	2.39	1,263	0	0.00	486	0	0.00
**Southern Europe**																										
Portugal	Coimbra^a^	PCR	803	5	0.62	374	4	1.07	924	90	9.74	460	38	8.26	1,084	19	1.75	469	8	1.71	161	0	0.00	69	0	0.00
Greece	Athens (A)^a^	*IgM ELISA*	*212*	*19*	*8.96*	*92*	*9*	*9.78*	*236*	*51*	*21.61*	*125*	*32*	*25.60*	*250*	*65*	*26.00*	*118*	*28*	*23.73*	*167*	*35*	*20.96*	*73*	*15*	*20.55*
		*IgG ELISA*	*212*	*44*	*20.75*	*92*	*19*	*20.65*	*236*	*29*	*12.29*	*125*	*13*	*10.40*	*250*	*37*	*14.80*	*118*	*16*	*13.56*	*167*	*41*	*24.55*	*73*	*19*	*26.03*
	Athens (B)^a^	*IgM ELISA*	*185*	*9*	*4.86*	*90*	*3*	*3.33*	*181*	*15*	*8.29*	*87*	*6*	*6.90*	*231*	*27*	*11.69*	*106*	*14*	*13.21*	*172*	*14*	*8.14*	*90*	*8*	*8.89*
		*IgG ELISA*	*185*	*59*	*31.89*	*90*	*25*	*27.78*	*181*	*88*	*48.62*	*87*	*46*	*52.87*	*231*	*92*	*39.83*	*106*	*46*	*43.40*	*172*	*44*	*25.58*	*90*	*20*	*22.22*
Slovenia	Ljubljana	PCR	530	19	3.58	708	7	0.99	745	119	15.97	857	75	8.75	1,326	402	30.32	1,382	218	15.77	320	14	4.38	528	8	1.52
**Asia**																										
**Western Asia**																										
Israel	Jerusalem	PCR	256	17	6.64	573	19	3.32	337	39	11.57	610	33	5.41	364	29	7.97	760	25	3.29	216	0	0.00	275	0	0.00
**Eastern Asia**																										
Japan	Kurashiki City (Okayama)^a^	PCR	30	4	13.33	16	2	12.50	64	14	21.88	26	5	19.23	34	3	8.82	15	1	6.67	5	0	0.00	5	0	0.00
	Tokyo	Rapid antigen test	25	NA	NA	52	33	63.46	80	25	31.25	60	9	15.00	420	22	5.24	180	14	7.78	60	3	5.00	60	1	1.67
Taiwan	Taoyuan^a^	PCR	116	20	17.24	56	11	19.64	159	63	39.62	77	31	40.26	204	131	64.22	113	71	62.83	44	5	11.36	16	0^b^	0.00
**South-eastern Asia**																										
Singapore	Singapore^a^	PCR	4,212	387	9.19	NA			8,765	307	3.50	NA			15,860	613	3.87	NA			8,835	33	0.37	NA		
**South Asia**																										
India	New Delhi	*IgM ELISA*	*159*	*12*	*7.55*	*30*	*7*	*23.33*	*207*	*7*	*3.38*	*105*	*8*	*7.62*	*113*	*14*	*12.39*	*67*	*7*	*10.45*	*84*	*13*	*15.48*	*49*	*5*	*10.20*
**America**																										
**Northern America**																										
United States	Chicago^a^	PCR	3,818	10	0.26	1,892	3	0.16	3,873	21	0.54	1,814	15	0.83	4,653	39	0.84	2,258	21	0.93	1,589	2	0.13	735	0	0.00
**Caribbean**																										
Cuba	National surveillance	PCR	535	12	2.24	398	6	1.51	38	1	2.63	25	0	0.00	497	15	3.02	385	6	1.56	0	NA	NA	0	NA	NA
**Oceania**																										
Australia	Darlinghurst (Sydney)	PCR	3,975	35	0.88	8,303	36	0.43	3,050	30	0.98	6,241	22	0.35	4,784	111	2.32	11,242	82	0.73	9,487	10	0.11	36,408	10	0.03
New Zealand	Auckland	PCR	154	11	7.14	252	10	3.97	167	8	4.79	475	13	2.74	226	22	9.73	401	21	5.24	561	3	0.53	1,219	3	0.25

COVID-19: coronavirus disease; ELISA: enzyme-linked immunosorbent assay; Ig: immunoglobulin; NA: not available; UN: United Nations.

^a^ ≥ 90% of data are from children and adolescents < 18 years of age.

^b^ Statistically significant difference in proportions of children/adolescents or females with positive tests between April 2020 and March 2021 and between April 2017 and March 2020 (Fisher's exact test, p < 0.05).

For serology only total test numbers of IgM considered. Entries in italics signify serological data (± PCR).

Grey backgrounds indicate local stay-at-home order (lockdown) periods. Another site from Germany (Homburg) did also provide PCR and serological data separately but numbers by month were not available.

Following the introduction of NPIs, the *M. pneumoniae* incidence by direct test methods decreased significantly from 8.61% ± 10.62 (mean of incidences from each site ± standard deviation) during April 2017 to March 2020 to 1.69% ± 3.30 in April 2020 to March 2021 (p < 0.01). The detection rates decreased with direct but not with indirect test methods (−93.51% vs +18.08%; p < 0.01) (Table 2). Although 27 sites reported also a reduction in total number of tests (–44.52% ± 24.61) in April 2020 to March 2021, seven sites showed an increase in total test numbers during the COVID-19 pandemic (because SARS-CoV-2 PCR was included in a multiplex panel that also contained *M. pneumoniae *PCR) (Table 2). In the year before the introduction of NPIs (April 2019 to March 2020), direct *M. pneumoniae* detections were significantly increased in several countries across UN regions compared with the period April 2018 to March 2019, which was indicative of an *M. pneumoniae* epidemic (Figure 2A).

Total duration of lockdown (82.80 days ± 55.73; range: 0–240) and school closure periods (84.05 days ± 56.33; range: 0–235) varied widely across countries. There was no correlation of the duration of lockdown or school closure periods with direct *M. pneumoniae* detection rates from April 2020 to March 2021. Several sites reported a longer duration of lockdown than school closure periods, which suggested that children returned to schools while lockdown continued for some time (Table 1). The re-opening of schools had no observable impact on the incidence of *M. pneumoniae* as direct detections remained remarkably low throughout the period April 2020 to March 2021. Detections were very low or absent even in countries where no school closures or official lockdowns were enforced (e.g. Japan, Taiwan; see Supplementary Figure S3 for *M. pneumoniae *detections in Asia).

### Macrolide resistance

**Figure 4:**
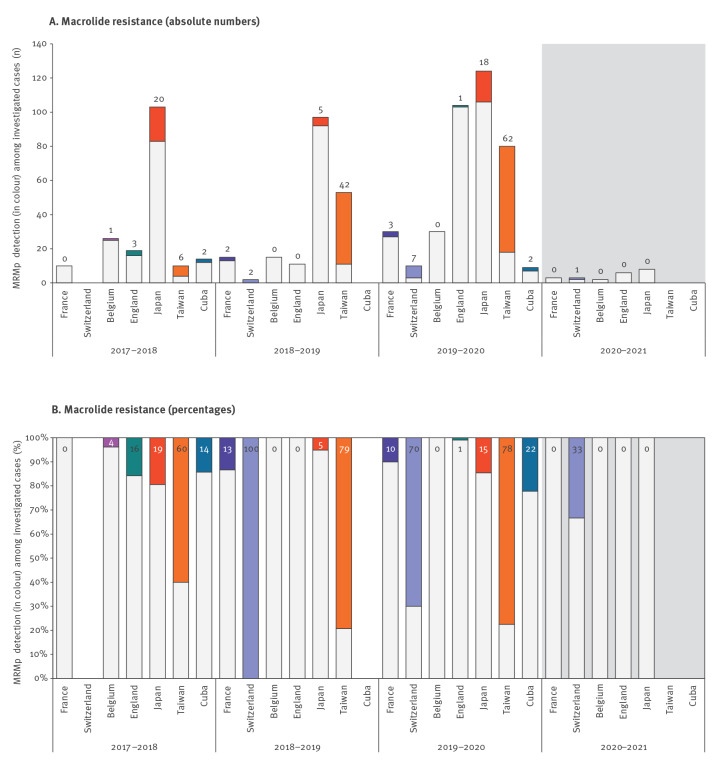
Macrolide-resistant *Mycoplasma pneumoniae* testing and detection in different countries across the world, April 2017–March 2021 (n = 784)

As a consequence of the significant decrease in *M. pneumoniae* detections after the introduction of NPIs, only few cases were investigated for macrolide resistance. In total, seven sites from Europe, Asia and America reported MRMp rates from April 2017 to March 2021 (Table 4). Macrolide resistance determination was reported as part of national surveillance of positive samples (Japan, Cuba) or only on positive samples identified at the reference laboratory and/or upon physician request. The MRMp detections among investigated cases are shown as absolute numbers in Figure 4A and as percentages in Figure 4B. The highest MRMp rate was found in Taiwan from April 2018 to March 2019 with 42 of 53 isolates. The national surveillance from Japan contributed the greatest number of strains investigated for macrolide resistance. Overall, MRMp was detected in one of 22 investigated cases from April 2020 to March 2021 and in 176 of 762 (23.10%) from April 2017 to March 2020 (p = 0.04).

**Table 4:** Macrolide-resistant *Mycoplasma pneumoniae* testing and detection rates per year, April 2017–March 2021 (n = 784)

UN region and country	City or region	Macrolide resistance determination (reference)	April 2017–March 2018			April 2018–March 2019			April 2019–March 2020			April 2020–March 2021 (COVID-19 pandemic)			Difference in detection rate (%) pre-pandemic vs COVID-19 pandemic^a^	*P* ^b^
			Total tests (*N*)	Positive tests (*n*)	Detection rate (%)	Total tests (*N*)	Positive tests (*n*)	Detection rate (%)	Total tests (*N*)	Positive tests (*n*)	Detection rate (%)	Total tests (*N*)	Positive tests (*n*)	Detection rate (%)		
**Europe**																
**Western Europe**																
France	Bordeaux	[48]	10	0	0.00	15	2	13.33	30	3	10.00	3	0	0.00	**–100.00**	1.00
Switzerland	*Zurich (A + B^c^)^d^*	[50]	*0*	NA	NA	*2*	*2*	*100.00*	*10*	*7*	*70.00*	*3*	*1*	*33.33*	**–*55.56***	*0.24*
Belgium	Antwerp, Leuven (national reference laboratory)	[48]	26	1	3.85	15	0	0.00	30	0	0.00	2	0	0.00	**–100.00**	1.00
England	National reference laboratory^e^	[55]	19	3	15.79	11	0	0.00	104	1	0.96	6	0	0.00	**–100.00**	1.00
**Asia**																
**Eastern Asia**																
Japan	National surveillance	[58]	103	20	19.42	97	5	5.15	124	18	14.52	8	0	0.00	**–100.00**	0.60
Taiwan	Taoyuan*^c^*	[59]	10	6	60.00	53	42	79.25	80	62	77.50	0	NA	NA	NA	NA
**America**																
**Caribbean**																
Cuba	National surveillance	[60]	14	2	14.29	0	NA	NA	9	2	22.22	0	NA	NA	NA	NA

COVID-19: coronavirus disease; SD: standard deviation; MRMp: macrolide-resistant *Mycoplasma pneumoniae*; NA: not applicable; UN: United Nations.

^a^ Difference in detection rate between April 2017 and March 2020 (mean positive/total tests across the 3 years) and April 2020 and March 2021 (absolute number positive/total tests). Percentages showing a reduction in detection rate are indicated in bold.

^b^ Proportions of positive/total tests from April 2020 to March 2021 were compared with total numbers from April 2017 to March 2020 by Fisher's exact test.

^c^ ≥ 90% of data are from children and adolescents < 18 years of age.

^d^ Macrolide resistance determination only upon physician's request in case of clinically suspected MRMp infection. Data reported for both sites from Zurich (A + B).

^e^ Period of enhanced surveillance from 1 October 2019 to 30 March 2020.

Entries in italics signify macrolide resistance determination only upon physician’s request in case of clinically suspected MRMp infection.

MRMp: Macrolide-resistant *Mycoplasma pneumoniae.*

The coloured parts of the bar graph with numbers represent absolute numbers or proportions of MRMp detection (the colours correspond with colours for sites in Figure 2). Data derived from the COVID-19 pandemic (April 2020–March 2021) are indicated by a grey background. Japan and Cuba reported national MRMp surveillance data (Table 4). Macrolide resistance determination in Switzerland was performed only upon request from a physician (in case of clinically suspected MRMp infection).

## Discussion

This global survey showed that all countries experienced a decrease in *M. pneumoniae* incidence by direct test methods in April 2020–March 2021, relative to the previous three years. This decline corresponded with the timing of the implementation of NPIs against COVID-19 in March 2020 in each country. We also observed a decrease in MRMp rates in April 2020 to March 2021. The MRMp rates before the COVID-19 pandemic were lower in Europe than in America or Asia, consistent with previous reports [11].

A reduction in *M. pneumoniae* detections after the introduction of NPIs was observed with direct test methods such as PCR but not with serology. This effect could be explained by the long-lasting nature of antibodies against *M. pneumoniae*. *Mycoplasma pneumoniae*-specific antibodies (IgM and IgG) persist for months to years after infection, and significantly longer than *M. pneumoniae* DNA in the upper respiratory tract [30,31]. Based on these kinetics, we would expect a decline in positive IgM serology in the second year of the COVID-19 pandemic, but not necessarily in IgG serology as *M. pneumoniae*-specific IgG antibodies can persist lifelong [30]. There is also the possibility of false-positive results caused by limited assay performance [32] as serological detections are reported from single-sample serology, which was in most cases not confirmed by the detection of a significant antibody level change in convalescent sera. In addition, PCR and serology (IgM and IgG) can be positive in asymptomatic carriers [11]. The detection of specific antibody-secreting cells by enzyme-linked immunospot (ELISpot) assay may allow for differentiation between infection and carriage [24], and a combination of clinical features and biomarkers can help identify patients at high risk for *M. pneumoniae* community-acquired pneumonia [15]. However, no clinical features were reported in this study and cases were defined by local practice.

Our findings are in line with several reports about a worldwide reduction in infections with respiratory and gastrointestinal pathogens after the introduction of NPIs [2,3,5-7,33-37]. The incidence of invasive bacterial diseases caused by *Streptococcus pneumoniae*, *Haemophilus influenzae*, and *Neisseria meningitidis* that are transmitted via the respiratory route were also considerably reduced during the early months of the COVID-19 pandemic [38]. The interruption of direct person-to-person transmission was suspected to be the most plausible explanation for the reduction in respiratory infections. These remained low even after the re-opening of schools, except for rhinovirus [6,39-41].

Direct detections of *M. pneumoniae* between April 2020 and March 2021 were significantly below levels of non-epidemic periods of *M. pneumoniae* across countries despite widely differing lockdown or school closure periods, and even in countries where no official lockdowns or school closures were enforced. This suggests that the observed low *M. pneumoniae* incidence may be explained by the continuation of NPIs such as personal protective and physical distancing measures. Other factors that may be involved in restricting *M. pneumoniae* transmission are behavioural responses to the pandemic (e.g. limited mobility related to COVID-19) and change in healthcare utilisation (e.g. telemedicine visits). After the re-opening of schools, direct *M. pneumoniae* detections remained low. This was also observed at sites where lockdown and restrictions for the adult population continued while children returned to schools. Children have greater difficulty adhering to physical distancing and personal protective measures so that *M. pneumoniae* transmission may be less effectively prevented in schools than in the adult population. Unfortunately, we did not have information on the age distribution in children to look at the pre-school and school age groups separately. The low incidence despite the re-opening of schools might suggest that adults play a more important role in transmission of *M. pneumoniae* than previously thought. This is supported by the observed decrease in the proportion of children and adolescents with *M. pneumoniae* detection during the COVID-19 pandemic. Notably, there was no change in the proportion of females with* M. pneumoniae* infection before and during the COVID-19 pandemic. Reduced transmission by shielding of adults (regardless of school closures) was also discussed as possible reason for the decrease in invasive pneumococcal disease [38]. Interestingly, nasopharyngeal pneumococcal carriage in children was only slightly reduced during the first year of the COVID-19 pandemic and the reduction in invasive pneumococcal disease was therefore attributed to the suppression of specific respiratory viruses such as RSV and influenza, which are often implicated as co-pathogens with *S. pneumoniae* [42]. *Mycoplasma pneumoniae* is also frequently detected with other viruses in the upper respiratory tract [15,43-45], but the role of co-detections in *M. pneumoniae* respiratory disease remains unclear [44]. A direct biological effect of SARS-CoV-2 on *M. pneumoniae* by interference or interaction could be another explanation. To our knowledge, data supporting this hypothesis do not exist so far. Further, transient herd immunity from the recent epidemic period in April 2019–March 2020 in several countries in Europe and Asia could have led to a decreased *M. pneumoniae* incidence during the COVID-19 pandemic [12]. However, the incidence was also reduced in countries that had not experienced a recent epidemic (e.g. Norway).

The study has a number of limitations. Firstly, because of the variable reporting methods and testing criteria at each site, conclusions based on the analysis across countries must be considered with caution. Data obtained from a single hospital laboratory from a specific region may not be fully representative of the country as a whole. No information about catchment area and numbers of laboratories within regions were available. The study also lacks representation from Africa and South America (no survey response and/or no testing for *M. pneumoniae* reported). Secondly, defining study-wide case definitions and de-duplication criteria was not feasible given the heterogeneous nature of data collection between sites. De-duplication methodologies were therefore set at site level. Thirdly, as mentioned previously, serological detections were not confirmed by antibody changes in paired sera in most cases. Fourthly, analysis of the local clinical testing pathway for *M. pneumoniae* was not possible within this study. Decision-making to test or not to test with specific methodologies during the COVID-19 pandemic may have impacted which individuals and sites offered testing at which time. The number of tests increased in one fifth of the sites during the period April 2020 to March 2021 and also the incidence was significantly lower compared with the pre-pandemic period; hence, we do not believe that the overall reduction in *M. pneumoniae* detections can solely be accounted for by reduced testing. Nor was there an indication that *M. pneumoniae* testing was reduced because of shifting laboratory resources towards SARS-CoV-2 testing during the whole first year after the introduction of NPIs covered by this study. Finally, an overall survey response rate could not be calculated because of the widespread dissemination of the survey. Incomplete response to a survey can introduce a bias related to differences in incidence between the responders and the non-responders [21,46]. However, this risk seems minimal as our survey dealt with microbiological laboratory data and generated a large and varied sample [46].

This study is another example of how pandemic-focused public health measures may have prevented infections caused by other respiratory pathogens. The COVID-19 pandemic resulted in restrictive NPIs such as lockdowns and school closures, which are unsustainable in the longer term. The results of this study suggest that even less restrictive NPIs such as personal protective and physical distancing measures might have prevented transmission of *M. pneumoniae* in the community.

The study also highlights the importance of establishing international working groups to investigate pathogen epidemiology where surveillance systems are lacking. It underlines the need for an international case definition for infection with *M. pneumoniae* (detection method and clinical criteria). The influence of the detection method for epidemiological surveillance of *M. pneumoniae* is shown in the discrepancy between PCR and single-sample serology in this study. Serological surveillance of *M. pneumoniae* may be only accurate by using paired sera in order to detect a fourfold increase in IgG levels [11]. However, such procedures are time-consuming and are not useful for acute patient care. A more rapid response to public health measures may be obtained by surveillance of *M. pneumoniae* using PCR.

Finally, epidemiological surveillance should also include antimicrobial resistance testing of *M. pneumoniae*. This study represents the most comprehensive estimate of global resistance documented to date and is important for clinicians and infectious disease surveillance considering that macrolides remain the main global treatment option for children with *M. pneumoniae* infection.

## Conclusion

The results of this study from diverse geographical locations and healthcare settings suggest that the implementation of NPIs against COVID-19 probably restricted transmission of *M. pneumoniae*, leading to a significant reduction in *M. pneumoniae* infections in many countries across the world from April 2020 to March 2021. The retention of some NPIs after the COVID-19 pandemic e.g. improved hand hygiene, respiratory etiquette or physical distancing in the community, or the use of masks in health care institutions may help reduce the burden of *M. pneumoniae* infections. The large collaborative network established for this study allows to assess the resurgence of *M. pneumoniae* infections at a later time. 

## Supplementary Data

1237000 Supplement
