# Supplementary material for: Mycoplasma pneumoniae detections before and during the COVID-19 pandemic: results of a global survey, 2017 to 2021
Source: Euro Surveill. 2022 May 12;27(19):2100746. doi: 10.2807/1560-7917.ES.2022.27.19.2100746 (PMC9101966; doi:10.2807/1560-7917.ES.2022.27.19.2100746)

Supplementary material

This supplementary material is hosted by *Eurosurveillance* as supporting information alongside the article *[Mycoplasma pneumoniae detections before and during the COVID-19 pandemic: results of a global survey, 2017–2021]*, on behalf of the authors, who remain responsible for the accuracy and appropriateness of the content. The same standards for ethics, copyright, attributions, and permissions as for the article apply. Supplements are not edited by *Eurosurveillance* and the journal is not responsible for the maintenance of any links or email addresses provided therein.

Tables

Supplementary Table S1. Details of the survey.

ESCMID EUROPEAN SOCIETY OF CLINICAL MICROBIOLOGY AND INFECTIOUS DISEASES

MyCOVID ESGMAC survey Survey

Mycoplasma pneumoniae prevalence before and during the COVID-19 pandemic: the MyCOVID survey on behalf of the ESGMAC

Overview of the survey:
1) Details of the survey participant
2) Information on laboratory and area
3) Local COVID-19 information
4) Information on test method for *Mycoplasma pneumoniae* detection
5) *Mycoplasma pneumoniae* test numbers
6) *Mycoplasma pneumoniae* macrolide resistance

General information:
– Please collect data about *Mycoplasma pneumoniae* detection and test numbers before entering the survey.
– The completion of the survey takes **20 min** (if data are on hand).
– There is no need to answer all of the questions. Please enter "**NA**" (not applicable) if no data / information exists.
– You will be able to revisit the survey to check and adjust your answers.
– It is also possible to send data / information via e-mail to patrick.meyer@kispi.uzh.ch and we will complete the survey for you.

1) Details of the survey participant

Last name		
First name		
Academic title		
Profession		
Affiliation		
E-Mail		
Consent to publish the data by the ESGMAC	Yes	No
Consent to be listed as survey participant with name and affiliation in the acknowledgment section of the publication	Yes	No
Ethical approval for data submission required according to local regulations	Yes	No
If yes, please add ethics committee and project number (if available)		

2) Information on laboratory and area

Type of laboratory / system	Hospital / clinical laboratory	Reference laboratory	Surveillance system
Name of laboratory / system			
Link (URL) to the laboratory / system			
If hospital / clinical laboratory, please add type of affiliated hospital	Primary	Secondary	Tertiary
City and post / zip code	City:	Post / zip code:	
Country			
Comments			

3) Local COVID-19 information

Please approve dates for your national pandemic lockdowns at https://en.wikipedia.org/wiki/COVID-19_lockdowns ("Table of pandemic lockdowns")	Correct	Incorrect
If incorrect, please add information		
School closure duration in months from 01.04.2020–31.03.2021		

4) Information on test method for *Mycoplasma pneumoniae* detection

Method	Nucleic acid amplification	Antigen detection	Serology	Other
Technique (e.g., multiplex PCR)				
Detection (e.g., microarray)				
Product (e.g., FilmArray Respiratory Panel)				
Company (e.g., bioMérieux/Biofire Diagnostics)				
Literature reference (if available)				
Comments				

5) *Mycoplasma pneumoniae* test numbers

**01.04.2020–31.03.2021 (pandemic)**	**Positive tests:**						**Total tests:**					
– Children (<18 years)	Positive tests:						Total tests:					
– Sex (female)	Positive tests:						Total tests:					
– Positive tests by month	Apr:	May:	Jun:	Jul:	Aug:	Sep:	Oct:	Nov:	Dec:	Jan:	Feb:	Mar:
Comments												

**01.04.2019–31.03.2020**	**Positive tests:**						**Total tests:**					
– Children (<18 years)	Positive tests:						Total tests:					
– Sex (female)	Positive tests:						Total tests:					
– Positive tests by month	Apr:	May:	Jun:	Jul:	Aug:	Sep:	Oct:	Nov:	Dec:	Jan:	Feb:	Mar:
Comments												

**01.04.2018–31.03.2019**	**Positive tests:**						**Total tests:**					
– Children (<18 years)	Positive tests:						Total tests:					
– Sex (female)	Positive tests:						Total tests:					
– Positive tests by month	Apr:	May:	Jun:	Jul:	Aug:	Sep:	Oct:	Nov:	Dec:	Jan:	Feb:	Mar:
Comments												

**01.04.2017–31.03.2018**	**Positive tests:**						**Total tests:**					
– Children (<18 years)	Positive tests:						Total tests:					
– Sex (female)	Positive tests:						Total tests:					
– Positive tests by month	Apr:	May:	Jun:	Jul:	Aug:	Sep:	Oct:	Nov:	Dec:	Jan:	Feb:	Mar:
Comments												

6) *Mycoplasma pneumoniae* macrolide resistance

Method		
**01.04.2020–31.03.2021 (pandemic)**	Macrolide resistance detection:	Testing for macrolide resistance:
**01.04.2019–31.03.2020**	Macrolide resistance detection:	Testing for macrolide resistance:
**01.04.2018–31.03.2019**	Macrolide resistance detection:	Testing for macrolide resistance:
**01.04.2017–31.03.2018**	Macrolide resistance detection:	Testing for macrolide resistance:
Comments		

Supplementary Table S2. Reporting characteristics, de-duplication and exclusion criteria by participating sites.

UN region and country	City or region	Laboratory affiliation	Ethical approval for data submission required according to local regulations	De-duplication	Exclusion
**Europe**					
**Western Europe**					
- France	Bordeaux	Laboratory of Bacteriology, Pellegrin Hospital, Bordeaux, France	No**	All the numbers provided are from episodes of patients being tested. This means that if a patient has been tested more than once within 90 days, only the first positive or, when the patient only tested negative, the first negative test has been retained for analysis.	None given
- Switzerland	Geneva	Bacteriology Laboratory, Division of Laboratory Medicine, Department of Diagnostics, Geneva University Hospitals, Geneva, Switzerland	No**	None given	None given
	Lausanne	Clinique de La Source - Laboratories, Lausanne, Switzerland	No**	None given	None given
	Bern*	Institute for Infectious Diseases, University of Bern, Bern, Switzerland.	No**	None given	None given
	Lucerne*	Department of Laboratory Medicine, Cantonal Hospital Lucerne, Switzerland	No**	None given	No total test number available for 2017-04-01 to 2018-03-31.
	Bellinzona	Department of Microbiology, Bellinzona, Switzerland	No**	None given	None given
	Zurich (A)	Institute of Medical Microbiology, University of Zurich, Zurich, Switzerland	No**	None given	None given
	Zurich (B)*	Microbiology Laboratory, Division of Infectious Diseases and Hospital Epidemiology, University Children’s Hospital Zurich, Zurich, Switzerland	No**	All the numbers provided are from episodes of patients being tested. This means that if a patient has been tested more than once within 90 days, only the first positive or, when the patient only tested negative, the first negative test has been retained for analysis.	None given
	St. Gallen*	Center for Laboratory Medicine, St Gallen, Switzerland	No**	None given	None given
	Aarau	Department of Clinical Microbiology, Institute of Laboratory Medicine, Kantonsspital Aarau, Aarau, Switzerland	No**	None given	None given
	Basel (A)	Clinical Bacteriology and Mycology, University Hospital Basel, Basel, Switzerland	No**	None given	None given
	Basel (B)*	Department of Infectious Diseases and Vaccinology, University of Basel Children’s Hospital, Basel, Switzerland	No**	None given	None given
- Germany	Homburg	Center for Infectious Diseases, Institute of Medical Microbiology and Hygiene, Saarland University, Homburg, Germany	No**	None given	None given
	Düsseldorf	Institute for Medical Microbiology and Hospital Hygiene, Heinrich Heine University Düsseldorf, Düsseldorf, Germany	No**	None given	None given
	Saxony	Landesuntersuchungsanstalt Sachsen, Dresden, Germany	No**	None given	No total test numbers available and/or reported.

- Belgium	Antwerp, Leuven (national reference laboratory)	National reference laboratory, Universitair Ziekenhuis Antwerpen and UZ Leuven/KU Leuven, Belgium<br><br>Additional data analysis by the national public health institute Sciensano, Brussels, Belgium	No**	All the numbers provided are from episodes of patients being tested. This means that if a patient has been tested more than once within 90 days, only the first positive or, when the patient only tested negative, the first negative test has been retained for analysis.	None given
	National surveillance	Belgian Sentinel Network of Laboratories (SNL), coordinated by the national public health institute Sciensano, Brussels, Belgium	No**	All the numbers provided are from episodes of patients being tested. This means that if a patient has been tested more than once within 90 days, only the first positive or, when the patient only tested negative, the first negative test has been retained for analysis.	The data of the SNL include "Mycoplasma-like illness" syndromes cases confirmed by direct detection in nasopharyngeal or oropharyngeal swabs, or lower respiratory tract specimens. Cases based on serology have been excluded. No total test numbers available and/or reported.
- The Netherlands	Rotterdam	Medical Microbiology and Infectious Diseases, Erasmus Medical Center, Rotterdam, The Netherlands	No**	None given	No test and detection numbers available for 2017-04-01 to 2018-03-31.
**Northern Europe**					
- England	National reference laboratory	Bacteriology reference department (BRD), Public Health England, London, England, United Kingdom	No**	Duplicates have been removed in each period (April 1 – March 30). Duplicate samples across the four periods were classified as separate episodes. The first sample of duplicates was retained. Internal and external quality assessment samples.	None given
- Denmark	National surveillance	The Danish Microbiology Database (MiBa)	Yes (available)	At individual level only the first mycoplasma positive test result within a season is included. If several negative mycoplasma tests are available per individual, only one negative mycoplasma test is included/counted per week.	The first time an individual test positive for mycoplasma all subsequent negative and positive mycoplasma tests are excluded.
- Finland	Turku	Department of Clinical Microbiology, Turku University Hospital, Turku, Finland	No**	None given	None given
	National surveillance	National Infectious Disease Register (IDR) maintained by the Finnish National Institute for Health and Welfare (THL)	No**	None given	No total test numbers available and/or reported.
- Norway	Trondheim	Department of Medical Microbiology, St. Olavs Hospital, Trondheim University Hospital, Trondheim, Norway	Yes (available)	None given	None given
**Southern Europe**					
- Portugal	Coimbra*	Hospital Pediátrico, Centro Hospitalar e Universitário de Coimbra, Coimbra, Portugal	Yes (available)	None given	None given
- Greece	Athens (A)*	Department of Microbiology & Serology, P. & A. Kyriakou Children's Hospital, Athens, Greece	No**	None given	None given
	Athens (B)*	Microbiology Department, "Agia Sofia" Children's Hospital, Athens, Greece	No**	None given	None given
- Slovenia	Ljubljana	Institute of Microbiology and Immunology, Faculty of Medicine, University of Ljubljana, Ljubljana, Slovenia	Yes (0120-244/2021/3)	Duplicate samples from the same patients were included as a single case.	Serological data excluded.
**Asia**					
**Western Asia**					
- Israel	Jerusalem	Department of Clinical Microbiology and Infectious Diseases, Hadassah Hebrew University Medical Center, Jerusalem, Israel	Yes (HMO 460-12)	None given	None given
**Eastern Asia**					
- Japan	Kurashiki City (Okayama)*	Kawasaki Medical School, Kurashiki-City, Okayama, Japan	Yes (3119-1)	None given	None given
	Tokyo[1]	Eiju General Hospital, Tokyo, Japan	Yes (2015-8)	None given	No positive test numbers reported by month.
- Taiwan	Taoyuan*	Chang Gung Memorial Hospital, Taoyuan, Taiwan	Yes (201900420A3C502)	None given	None given

**South-eastern Asia**					
- Singapore	Singapore*	Department of Pathology and Laboratory Medicine, KK Women's and Children's Hospital, Singapore	No**	None given	None given
**South Asia**					
- India	New Delhi	Special Bacterial Pathogen Laboratory, Department of Microbiology, All India Institute of Medical Sciences, New Delhi, India	Yes (available)	None given	None given
**America**					
**Northern America**					
- USA	Chicago*	Ann & Robert H Lurie Children's Hospital of Chicago, Chicago, IL, USA	Yes (available)	None given	None given
**Caribbean**					
- Cuba	National surveillance	National Reference Laboratory of Mycoplasmas, Tropical Medicine Institute "Pedro Kourí", Center for Research, Diagnosis and Reference, La Habana, Cuba	Yes (1901074)	None given	None given
**Oceania**					
- Australia	Darlinghurst (Sydney)	Division of Microbiology, SydPath St Vincent's Hospital, Sydney, NSW, Australia	Yes (available)	None given	None given
- New Zealand	Auckland	Microbiology Department, LabPlus, Auckland District Health Board, Auckland, New Zealand	No**	Positive numbers in April 2020 include 1 patient with 2 positive tests; in October 2019, 2 patients with 2 positive tests; in January 2020, 1 patient with 2 positive tests; and in July 2018, 1 patient with 2 positive tests.	None given

* ≥90% of data are from children <18 years of age. ** Survey participants confirmed that ethical review and approval is not required for aggregated and anonymized data according to local regulations.

Figures

Supplementary Figure S1. Detection of *M. pneumoniae* in Western Europe by PCR (April 2017–March 2021). Gray backgrounds indicate local stay-at-home order (“lockdown”) periods.

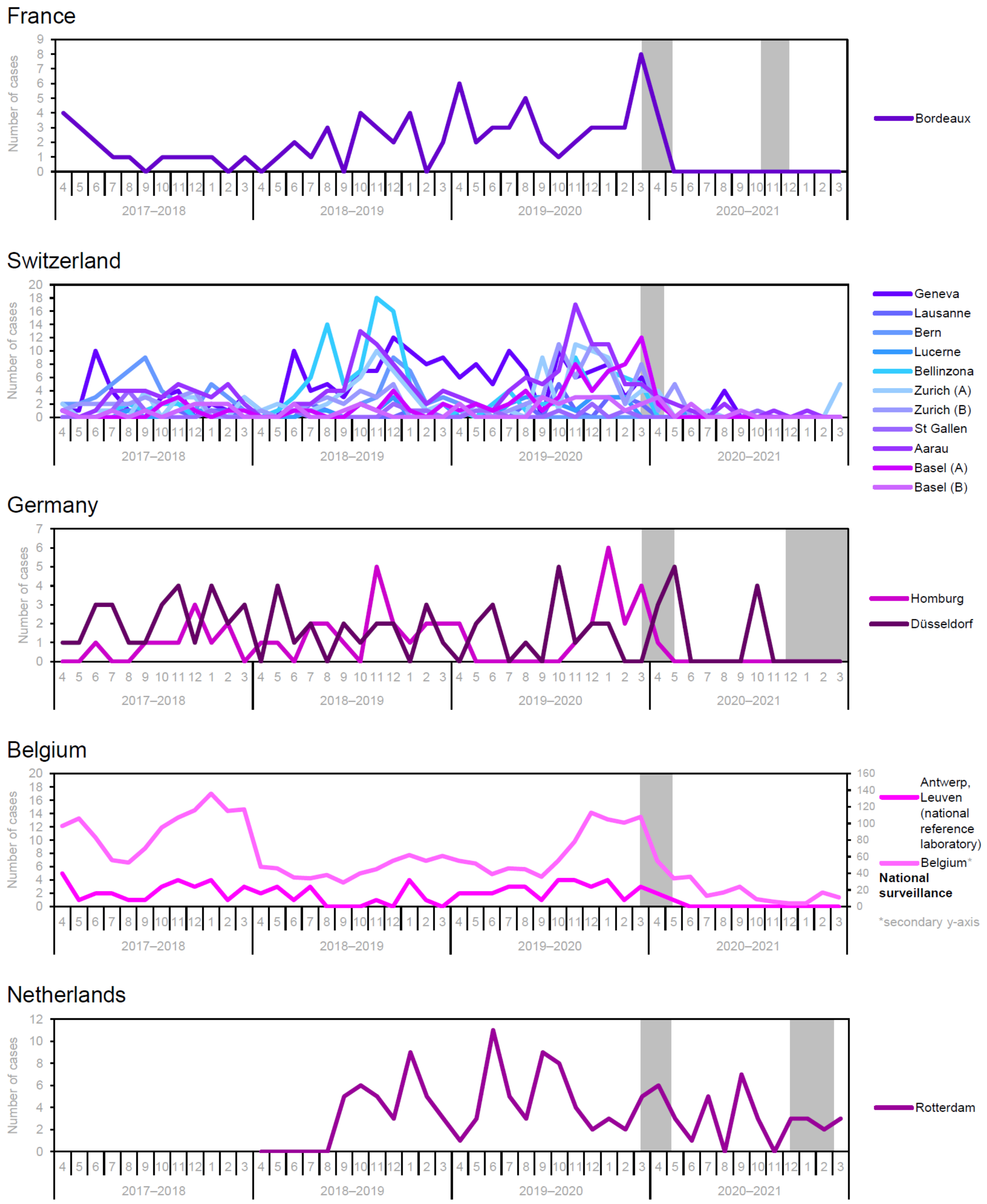

Supplementary Figure S2. Detection of *M. pneumoniae* in Northern and Southern Europe by PCR (April 2017–March 2021). Gray backgrounds indicate local stay-at-home order (“lockdown”) periods.

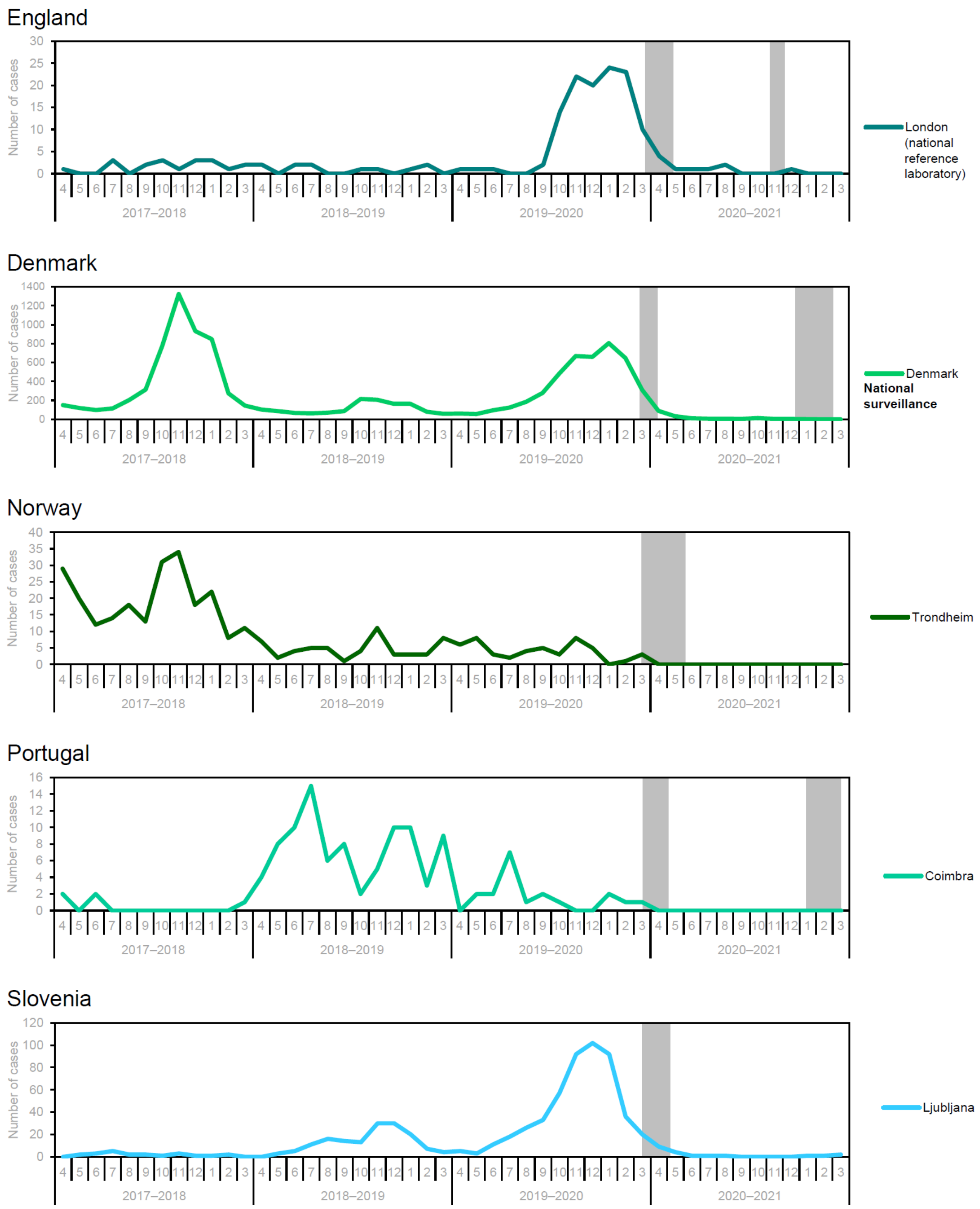

Supplementary Figure S3. Detection of *M. pneumoniae* in Asia by PCR (April 2017–March 2021). Gray backgrounds indicate local stay-at-home order (“lockdown”) periods.

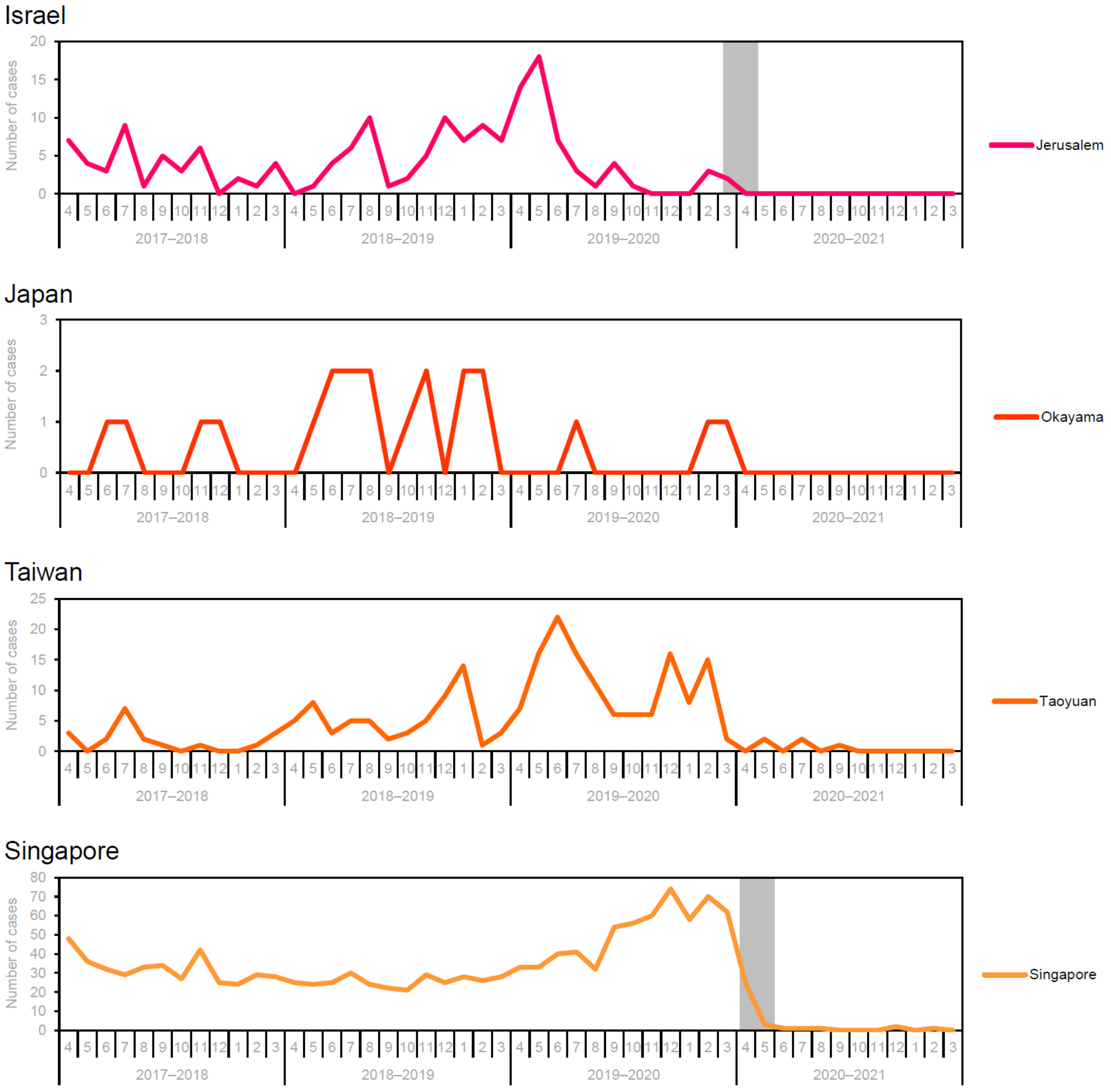

Supplementary Figure S4. Detection of *M. pneumoniae* in America and Oceania by PCR (April 2017–March 2021). Gray backgrounds indicate local stay-at-home order (“lockdown”) periods.

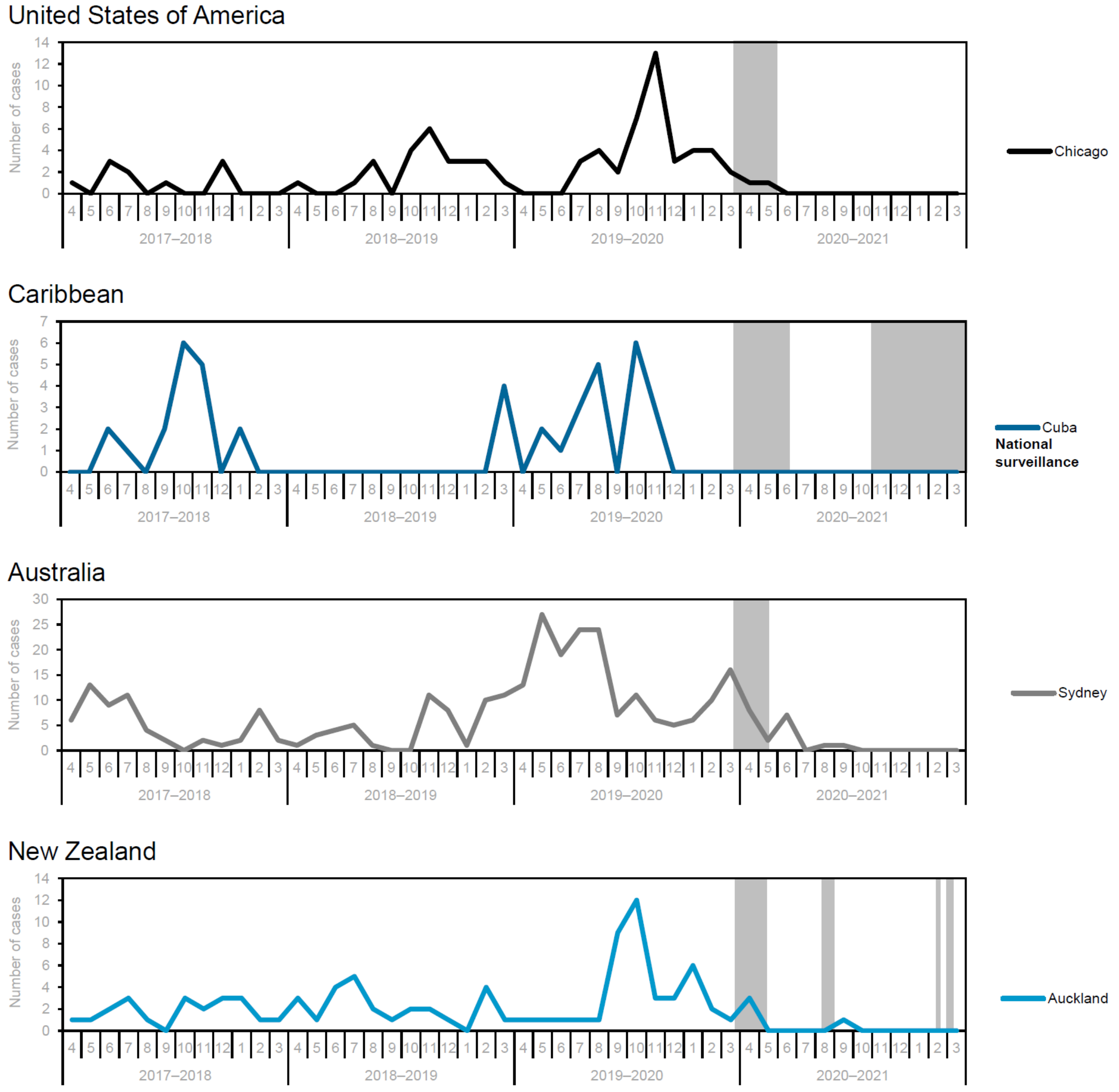

Supplementary Figure S5. Detection of *M. pneumoniae* by exclusively serology (April 2017–March 2021). Gray backgrounds indicate local stay-at-home order ("lockdown") periods. Data from sites which provided serology in addition to PCR are shown in Figure 3.

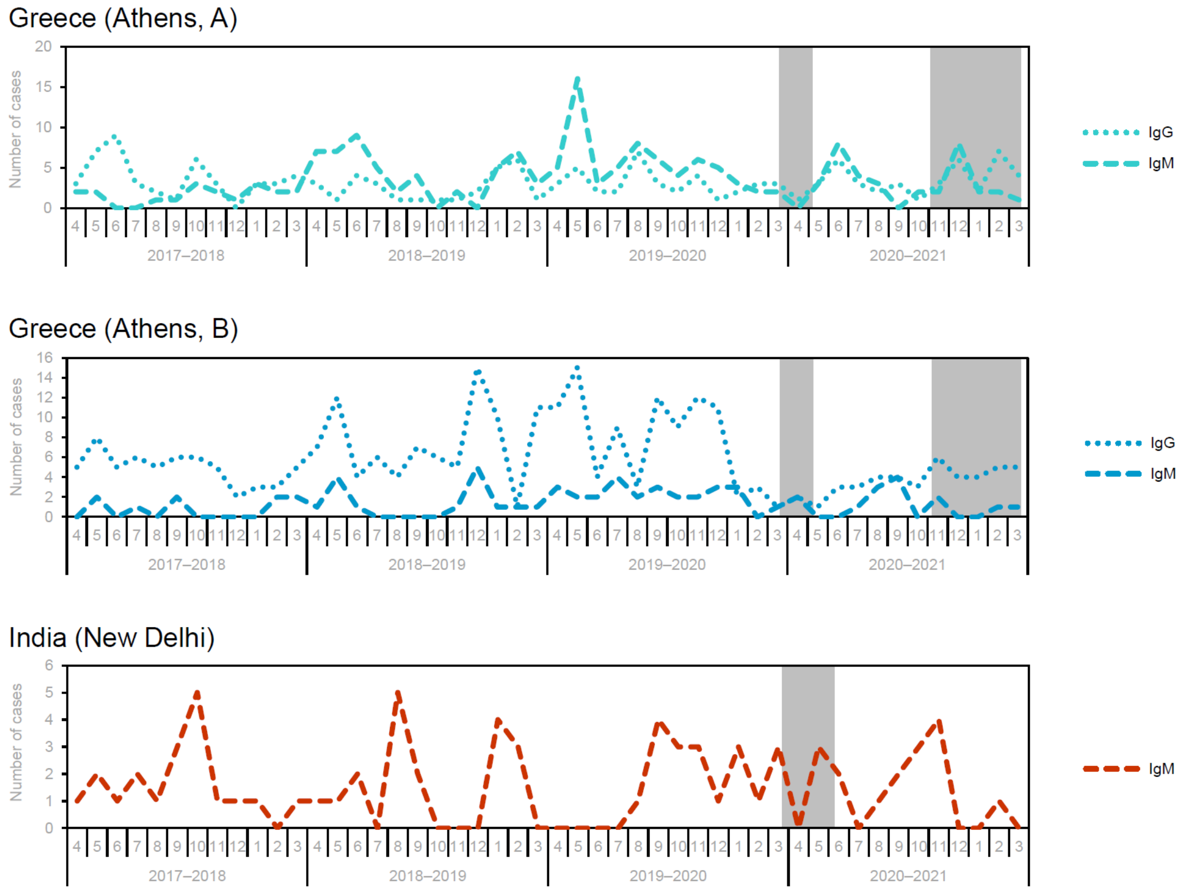

Supplementary Figure S6. Detection of *M. pneumoniae* by combination of direct and indirect test methods (no distinction possible between detection methods, but predominantly serology) (April 2017–March 2021). Gray backgrounds indicate local stay-at-home order ("lockdown") periods.

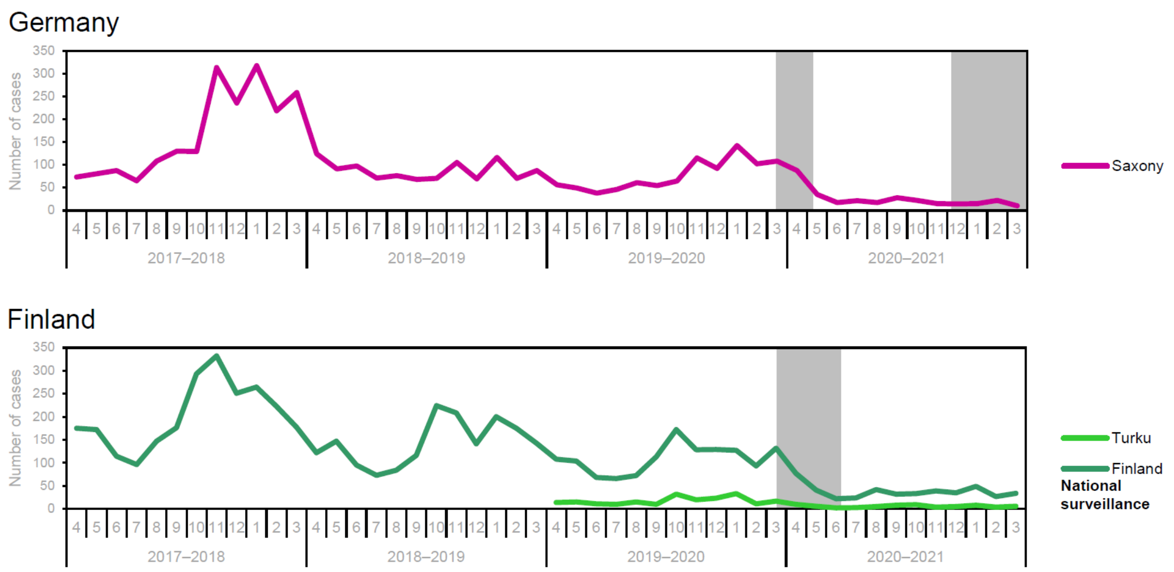

Supplement: Supplement [file 21-00746_MEYER-SAUTEUR_Supplement.pdf]
